# Ferrets as a model for tuberculosis transmission

**DOI:** 10.3389/fcimb.2022.873416

**Published:** 2022-08-16

**Authors:** Tuhina Gupta, Naveen Somanna, Thomas Rowe, Monica LaGatta, Shelly Helms, Simon Odera Owino, Tomislav Jelesijevic, Stephen Harvey, Wayne Jacobs, Thomas Voss, Kaori Sakamoto, Cheryl Day, Christopher Whalen, Russell Karls, Biao He, S. Mark Tompkins, Abhijeet Bakre, Ted Ross, Frederick D. Quinn

**Affiliations:** ^1^ Department of Infectious Diseases, College of Veterinary Medicine, University of Georgia, Athens, GA, United States; ^2^ Molecular Analytics R&D, GlaxoSmithKline Vaccines, Rockville, MD, United States; ^3^ Influenza Division, National Center for Immunization and Respiratory Diseases, Centers for Disease Control and Prevention, Atlanta, GA, United States; ^4^ Department of Comparative Biomedical Sciences, School of Veterinary Medicine, Louisiana State University, Baton Rouge, LA, United States; ^5^ Animal Resources Program, University of Georgia, Athens, GA, United States; ^6^ Merck Research Laboratories, West Point, PA, United States; ^7^ Department of Pathology, College of Veterinary Medicine, University of Georgia, Athens, GA, United States; ^8^ Department of Microbiology and Immunology, Emory University School of Medicine, Atlanta, GA, United States; ^9^ Department of Epidemiology and Biostatistics, College of Public Health, University of Georgia, Athens, GA, United States

**Keywords:** *mycobacterium*, tuberculosis, ferret, transmission, animal model

## Abstract

Even with the COVID-19 pandemic, tuberculosis remains a leading cause of human death due to a single infectious agent. Until successfully treated, infected individuals may continue to transmit *Mycobacterium tuberculosis* bacilli to contacts. As with other respiratory pathogens, such as SARS-CoV-2, modeling the process of person-to-person transmission will inform efforts to develop vaccines and therapies that specifically impede disease transmission. The ferret (*Mustela furo*), a relatively inexpensive, small animal has been successfully employed to model transmissibility, pathogenicity, and tropism of influenza and other respiratory disease agents. Ferrets can become naturally infected with *Mycobacterium bovis* and are closely related to badgers, well known in Great Britain and elsewhere as a natural transmission vehicle for bovine tuberculosis. Herein, we report results of a study demonstrating that within 7 weeks of intratracheal infection with a high dose (>5 x 10^3^ CFU) of *M. tuberculosis* bacilli, ferrets develop clinical signs and pathological features similar to acute disease reported in larger animals, and ferrets infected with very-high doses (>5 x 10^4^ CFU) develop severe signs within two to four weeks, with loss of body weight as high as 30%. Natural transmission of this pathogen was also examined. Acutely-infected ferrets transmitted *M. tuberculosis* bacilli to co-housed naïve sentinels; most of the sentinels tested positive for *M. tuberculosis* in nasal washes, while several developed variable disease symptomologies similar to those reported for humans exposed to an active tuberculosis patient in a closed setting. Transmission was more efficient when the transmitting animal had a well-established acute infection. The findings support further assessment of this model system for tuberculosis transmission including the testing of prevention measures and vaccine efficacy.

## Introduction

*Mycobacterium tuberculosis* is a persistent pathogen that continues to plague humanity. The World Health Organization (WHO) estimates that approximately nine million new infections and 1.5 million tuberculosis deaths occurred in 2018 (WHO, 2019). Although tuberculosis is treatable and preventable, current public health strategies have not fully controlled its proliferation, particularly in disease-endemic areas. To date, the WHO has focused on handling the global tuberculosis problem by vaccinating infants with the attenuated *M. bovis* Bacillus Calmette-Guérin (BCG) vaccine and subsequently treating patients who test positive by sputum smear-microscopy, tuberculin skin test (TST), interferon-gamma release assay (IGRA), or tests with anti-mycobacterial therapy, followed by tracing and testing of potential contacts. Unfortunately, there are many challenges to this program, including the increasing development of drug-resistant *M. tuberculosis* strains, the absence of a vaccine that confers sterilizing immunity or effectively protects adults from pulmonary disease, and diagnostic methodologies that are sufficiently sensitive or specific to detect the early stages of infection when bacterial numbers are low and more-effectively treated, especially among contacts of *M. tuberculosis* culture-positive individuals (Lee et al., 2019).

Modeling tuberculosis in animals is a challenge for a variety of reasons. Rodent models permit the study of some complex interactions between hosts and a variety of human and animal pathogenic agents. Mice and guinea pigs in particular are relatively inexpensive, use standard caging and equipment, and allow for the examination of multifactorial traits that may not be possible outside of a living host. In addition, they provide an initial framework for the subsequent evaluation of interventions and therapeutics in physiologically more-appropriate, large animal tuberculosis models and, ultimately, humans. However, there are significant limitations including lack of important correlations with human tuberculosis disease stages, such as latency (Orme & Ordway, 2016) and lack of animal-to-animal transmission. Alternatively, it is known that some larger animals, specifically cattle and non-human primates (NHPs), develop a spectrum of tuberculosis disease signs and pathology similar to humans, can effectively transmit tuberculous bacilli *via* the aerosol and oral routes, and are used to develop reagents for immunological studies (Wong et al., 2019). However, due to cost and a dearth of appropriate facilities, use of large animals or NHPs is not always feasible for routine controlled-research studies. To accelerate the understanding of human tuberculosis disease transmission processes, a more physiologically-relevant, small animal model with an appropriate reagent pool is needed.

The ferret (*Mustela furo*) has become the small animal model of choice for the study of transmission of influenza virus and other respiratory pathogens, and to examine vaccine efficacy (Koster et al., 2012; Wong et al., 2019). Though the ferret is not significantly more expensive than the rodent models and supplies are sufficient, the model has limitations, including the need for special caging and animal handling procedures, and the need for more immunological reagents. Pertinent to this study, the ferret has been shown to be susceptible to infection with a number of mycobacterial species including *M. bovis* (McCallan et al., 2011; Byrom et al., 2015), *M. celatum* (Piseddu et al., 2011), *M. genavense* (De Lorenzi et al., 2018), *M. avium* (Bezos et al., 2016), and other non-tuberculous mycobacteria (Pollock, 2012). Ferrets are evolutionarily related to other members of the carnivorous mammal (Mustelidae) family, including the European badger and New Zealand possum; these related species contribute to transmission of *M. bovis* to cattle and other large animals and potentially to humans in Great Britain, New Zealand, and elsewhere (Allen et al., 2018). In the present study, we assess infection of ferrets with *M. tuberculosis* bacilli and observe the development of human-like tuberculosis signs and pathology, define an intratracheal infectious dose that reproducibly causes disease, and provide convincing data demonstrating animal-to-animal transmission.

## Materials and methods

### Preparation of inoculum

*Mycobacterium tuberculosis* strain Erdman was a kind gift from Dr. Anne Lenaerts, Colorado State University. Bacteria were cultured as pellicles on Proskauer-Beck (PB) medium and stock aliquots frozen at -80°C. For intratracheal instillation, *M. tuberculosis* stock aliquots were thawed and diluted to the desired concentration in sterile 0.01 M phosphate-buffered saline (PBS), pH 7.4, and passed through a tuberculin syringe to disrupt bacterial clumps immediately before use.

### Ferrets

Spayed female or neutered male ferrets (3-8 months old) were obtained (Triple F Farms, Sayre, PA). After acclimatization, ferrets were implanted subcutaneously with IPTT-300 transponders (BioMedic Data Systems, Seaford, DE) for identification and routine body temperature monitoring. Prior to *M. tuberculosis* infection, blood, nasal wash, and throat swabs were collected. Animals were pair-housed in individually ventilated stainless steel cages (Allentown, Inc., Allentown, PA) and provided with ferret High Density Diet 5L14 (LabDiet, St. Louis, MO) and fresh water *ad libitum*. These studies were conducted in accordance with recommendations from the Guide for the Care and Use of Laboratory Animals and within a program accredited by the Association for Assessment and Accreditation of Laboratory Animal Care, International (AAALAC-I). All animal experiments were performed with approval of the Institutional Animal Care and Use Committee at the University of Georgia (NIH Animal Welfare Assurance Number: A3437-01). Infected ferret activity level and respiratory distress (e.g. coughing) were assessed daily, temperatures were measured minimally once per week, and weight was assessed weekly.

### Infections

In the initial infection experiment, ferrets were sedated by intramuscular (IM) injection of 0.2 mg/kg butorphanol (Torbugesic-SA, Zoetis, Inc., Parsippany-Troy Hills, NJ) 10 minutes prior to dosing with 8 mg/kg ketamine (Ketaset, Zoetis, Inc.) and 0.04 mg/kg dexmedetomidine (Dexdor, Orion Corp., Finland). Once anesthetized, animals were placed in dorsal recumbency within a Class II-A2 biosafety cabinet. To facilitate the insertion of the endotracheal tube (ET) (Rusche, Morrisville, NC), 1% lidocaine-HCl (Hi-Tech Pharmacal Co. Inc., Amityville, NY) was applied to the epiglottis, the vocal folds, and the end of the ET tube. For infection, 100-mm polyethylene tubing (BD, Intramedic, Radnor, PA), attached to a 20 G blunt needle, was passed through the ET tube. Ferrets (six per group) were then intratracheally infected with 1.0 ml of *M. tuberculosis* stock diluted in sterile saline followed by 2 ml of air. The three doses examined were low (1 – 5 x 10^1^), medium (1 – 5 x 10^2^), or high (5 – 10 x 10^3^) colony forming units (CFU), while the control ferret was similarly given a 1.0 ml intratracheal dose of PBS. After instillation, the anesthesia was reversed by IM injection of atipamezole 0.2 mg/kg (Antisedan, Zoetis, Inc.). All animals were housed inside an animal biosafety level-3 (ABSL-3) facility and monitored daily for clinical signs of disease. A detailed list of animal specimen samplings and times performed during the course of the study are provided in **Table 1**. At weeks 4 and 7 post infection, three animals from each group (low, medium, and high dose) were euthanized and various organs harvested. One-half of each lung was perfused with 10% buffered formalin and used for pathology. The other half of the lung was homogenized in 10 ml sterile Phosphate buffered saline (PBS), and 100 μl homogenates diluted and plated on 7H10 gtADS plates for viable count (CFU) assessments. For MGIT, one ml of tissue homogenate, nasal wash or throat swab were prepared in MGIT buffer according to the manufacturer’s instructions and 500 μl of each sample was added to the MGIT tubes. The half spleen and MLN were homogenized in five ml PBS and dilutions were plated on 7H10 gtADS plates. The timeline for the infection experiments is outlined in **Supplemental Figure 1**.

**Table 1:** Assessment Schedule for Infection and Transmission Studies.

Time	Assessment/Sample	Purpose/Method
Daily	• Temperature • Activity level • Cough, sneeze, respiratory distress	• Overall health status
Weekly	• Weight	
Monthly	• Blood • Nasal wash, throat swab**; • Feces**	• ELISA* • ELISA, CFU, PCR, MGIT • PCR
Mid-point	• Skin test • BAL^#^	• TST • CFU, MGIT • MGIT
Prior to necropsy	• See ‘monthly’ and ‘mid-point’ assessments	• See ‘monthly’ and ‘mid-point’ methods
Necropsy	• BAL^#^, trachea^&^, lung, spleen, liver, stomach**, lymph node	• ELISA, PCR, CFU, MGIT, histopathology

*Abbreviations: ELISA (enzyme-linked immunosorbant assay for cytokine/chemokine and antibody concentration assessments in fluids), CFU (M. tuberculosis colony-forming units), PCR (M. tuberculosis IS6110 PCR), MGIT (mycobacterial growth indicator tube culture), BAL (broncho-alveolar lavage), TST (tuberculin skin test).

**Throat swabs, feces and stomach only examined in the inital infection study.

**^#^** BAL only collected in the middle-high dose transmission study.

**^&^** Trachea examined in the initial infection and very-high dose transmission studies.

### Transmission

In the medium-high dose transmission experiment, four transmitter ferrets (TR1 - TR4) were intratracheally instilled with a medium- high dose (1 x 10^3^ CFU) of *M. tuberculosis* bacilli. After 24 hours, four naive sentinel ferrets (SE1 - SE4) were each pair-housed with the respective TR animal (e.g. SE1 with TR1). The ferret cages had perforated stainless steel floors to minimize fecal-oral transmission between animals. At week 17, SE4 unexpectedly died of unrelated causes after anesthesia, and a new naive sentinel ferret, SE5, was co-housed with TR4. At week 24, SE1 and SE2 were separated from TR1 and TR2, respectively. TR1 and TR2 were then co-housed with naive sentinels SE6 and SE7, respectively. SE1 and SE2 were co-housed in a separate cage. This study contained two naïve control ferrets, F9 and F10, maintained in a separate ABSL-2 facility. The timeline for the medium-high dose transmission study is outlined in **Supplemental Figure 1**.

In the very-high dose transmission experiment, we wanted to test both aerosol and direct transmission using special cages. In these cages, transmitters (TR-A1 through TR-A6) were separated from the aerosol sentinels by a perforated steel barrier. These six transmitters were intratracheally infected with a very-high dose (5 X 10^4^ CFU), and after five weeks, TR-A4 and TR-A5 had to be euthanized. Six aerosol sentinels (SE-A1- SE-A6) were introduced into the other side of the cage steel barrier while three direct sentinels (SE-D1 – SE-D3) were pair-housed with TR-A1 – TR-A3, respectively. This high dose study could only be performed for five additional weeks before it was ended. Three uninfected control ferrets were kept within the same ABSL-3 facility. The timeline for the very-high dose transmission study is outlined in **Supplemental Figure 1**.

Overall, strict procedures were followed to prevent aberrant cross-contamination between animals; SE animals were handled before TR animals, and work surfaces and handlers’ gloves were disinfected between interactions with each animal.

### Daily and weekly observable clinical signs

Hallmark symptoms of clinical tuberculosis in humans and other large mammals include weight loss, temperature spikes, lethargy, and respiratory distress including coughing. Because of the variables introduced in each of the three studies, including infection dose and study duration, data presentation formats particularly for weight measurements varied.

Juvenile ferrets, which naturally gain weight as they mature, were employed in all studies. In the infection study, because of the short study time, the infected and control animals did not lose weight, and thus differences in percent weight gain for each of the infection dose groups and uninfected control was reported (**Supplemental Figure 2A**). In the medium-high dose transmission study, again because of the use of juvenile ferrets and the transmitter infection dose provided, it was anticipated that like the initial infection experiments, the early weeks of this transmission study would see differences in weight gain. However, with the longer study period, it was postulated that eventual weight loss could be observed. Thus, weekly weight change as a fraction of maximal weight attained by each animal was calculated [(weekly weight/maximum weight)*100] (**Supplemental Figure 3A**). Lastly, in the very-high dose transmission study, weight loss over time was able to be assessed (**Supplemental Figures 5A**, **B**).

For temperature measurements, regardless of the study variables, spiking temperatures for each measurement point in the three studies were assessed in a similar fashion by subtracting the mean of the baseline temperature taken for all the animals in the study one week prior to the beginning of the study (days -7 to 0) from the temperature observed at the indicated time points (**Supplemental Figures 2B**, **3B**, **5C**, **D**) for each animal.

For changes in activity level and respiratory distress compared to uninfected controls, lethargy and increased coughing, respectively, were the primary assessments. It should be noted that lethargy and respiratory stress signs could only be observed while study personnel were in physical proximity to the animals, which was one or two times daily and lasting only a few minutes at a time for routine husbandry or data collection purposes. Thus, documented observations are likely an underrepresentation of the actual level of lethargy and respiratory signs. Future studies will employ a remote noise-activated camera to record these aspects.

### Preparation of tissue homogenates

Various sample tissues, including trachea, lung-associated lymph nodes, lungs, liver, stomach and spleen, were removed post mortem. Half of each lung from transmitters and sentinels was homogenized in total 6.5 ml sterile PBS in tubes with 1-mm, zirconia-silica beads by bead milling for 30 seconds in an Omni Bead Ruptor (Kennesaw, GA). The buffer volume per half-lung was decreased from 10 ml in the initial infection study to 6.5 ml. In this medium-high dose transmission study, since the ferrets in the transmission study showed less disease symptomology, and during necropsy fewer gross lung lesions were observed; thus, the 6.5 ml volume was the minimum that we could use in order to generate sufficiently homogenized lysates and maximize the likelihood of detecting the resident bacilli.As detailed below, 500 μl aliquots of the tissue homogenates were then inoculated into BACTEC MGIT culture medium to assess growth, 100 μl assayed by PCR to detect presence of the *M. tuberculosis* IS6110, and one ml of lung homogenate was diluted 1:5, and plated onto Middlebrook 7H10 agar to enumerate viable bacteria (CFU). In the very-high dose experiment, the half lungs of transmitters and sentinels due to the severe symptomology and transmitter lung lesions observed in this experiment were homogenized in 20 – 25 ml PBS, serially diluted and plated on 7H10 agar plates. All other organs in all three experiments were homogenized in five ml PBS, serially diluted and plated on 7H10 agar plates.

### Mycobacteria growth indicator tube analysis

For qualitative culture, non-invasive samples were collected, including nasal washes, throat swabs, stomach gavages, and feces from the cage pan, decontaminated using 0.075% (final volume) N-acetyl-L-cysteine/NaOH/citrate followed by centrifugation at 3,000 X *g* for 15 minutes. Pellets from these clinical samples, as well as the post-mortem tissue homogenates described above, were resuspended in 1 ml of 0.01 M sterile phosphate-buffered saline (pH 7.2), inoculated into BACTEC MGIT 960 culture tubes, and incubated for up to 45 days in a BACTEC 960 system (BD, East Rutherford, NJ). Suspect *M. tuberculosis* culture-positive samples were assessed using IS6110 PCR.

### Mycobacterial viable counts

For quantitative culture, dilutions of tissue homogenates, nasal washes, and bronchoalveolar lavages were made in PBS with 0.05% Tween 80 and plated onto Middlebrook 7H10 agar (Difco, Waltham, MA) supplemented with 0.5% glycerol (Fisher, Waltham, MA), 10% Albumin-Dextrose-Catalase (BBL, Waltham, MA), 0.05% Tween 80 (7H10gtADC), and 10 µg/ml cycloheximide (Sigma, St. Louis, MO) and incubated at 37°C for up to 6 weeks. *Mycobacterium tuberculosis* colonies were counted, and the total CFU per sample calculated. Colonies with *M. tuberculosis* morphology were subsequently confirmed by IS6110 PCR.

### Blood collection

Anesthetized ferrets were placed in ventral-dorsal recumbency, the thoracic area cleaned with an alcohol swab, and blood collected *via* the cranial vena cava. Serum was separated and samples frozen at −80°C until used.

### Nasal wash and throat swab

Ferrets were lightly anesthetized by intramuscular injection of the ketamine/dexmedetomidine cocktail detailed earlier. Sterile PBS (3 ml) was slowly instilled into each naris using a catheter attached to a 3-ml tuberculin syringe, and expelled fluid was collected in sterile sample containers. Additionally, upper airway throat samples were collected with PBS-moistened sterile swabs. All collected materials were stored at -80°C until processed. The nasal washes and swabs were thawed and plated on 7H10gtADC plates with 10 µg/ml cycloheximide. An additional 100 μl from each preparation was heat-inactivated at 99°C for 20 minutes, passed through Zymoclean columns and PCR-amplified using IS6110-specific primers.

### Broncho-alveolar lavage

Once anesthetized, the ferret tracheal opening was visualized using a laryngoscope and lidocaine applied. After five minutes, a sterile endotracheal tube was inserted. The correct placement was determined by visualization of fogging of the tube. Sterile saline was drawn in a 10 ml syringe and injected into the lungs. The contents were withdrawn by pulling back on the syringe plunger. The broncho-alveolar lavage (BAL) fluid collected was maintained at 4°C until processed for MGIT, bacterial culture, and PCR.

### Tuberculin skin test

The flanks of lightly anesthetized ferrets were clipped with a standard veterinary clipper. The TST was performed with 1 or 2 µg purified protein derivative or 1,000 IU Old Tuberculin batch OT76 (Statens Serum Institute) injected intradermally in different regions of each animal with PBS as a negative control. The erythema and induration at the injection sites were measured at 24, 48, and 72 hours. Tuberculin skin tests were read in a blinded, duplicate manner using the tuberculin scale provided by CDC. Two independent readings were taken at right angles to each other (longitudinal and transverse) at 24 hours post-administration by a single animal handler. The longest diameter of induration was reported as final. All reactions were monitored for necrosis (Dharmadhikari et al., 2011).

### Histopathology

In the initial infection experiment, ferrets were euthanized 4 or 7 weeks after *M. tuberculosis* infection. In the medium-high and very-high dose transmission experiments, study terminations were 27 weeks and 10 weeks, respectively, after the transmitter ferrets were infected. Half of each organ was harvested and multiple sections of each organ examined for histopathology by a board-certified veterinary pathologist, with a specific focus on granuloma characteristics that included: overall architectural appearance, the type of granuloma (caseous, non-necrotizing, suppurative, or mixed), distribution pattern (focal, multifocal, coalescing, or invasive), and cellular composition (the absence or presence of some level of neutrophils, eosinophils, lymphocytic cuff, mineralization, fibrosis, multinucleated giant cells, or epithelioid macrophages). Overall, lesions were evenly distributed within tissues, suggesting each portion is representative of the whole. A 0-to-3 scoring system developed previously (Gupta et al., 2018) to assess each of these criteria was applied. Lung sections were analyzed for approximate percentage of affected lung area, number, distribution, and severity of granulomas, as well as other features, such as percentage of granulomas composed of neutrophils, and severity of perivascular cuffing, vasculitis, interstitial pneumonia, and pleuritis.

### IS6110 PCR

Volumes (50 -100 μl) of nasal washes, throat swabs, and homogenates of lung, lymph node, spleen, and liver prepared as described earlier were heat-inactivated, DNA purified using Zymoclean tubes (Zymo Research, Irvine, CA), and PCR-amplified using *M. tuberculosis* IS6110-specific primers. Bacterial colonies obtained on 7H10gtADC agar after plating organ homogenates, nasal washes, and throat swabs were assayed by PCR (95°C for five minutes; 95°C for 30 seconds, 60°C for one minute, 72°C for one min; 72°C for 10 min) with primers IS6110F (5′-CCT ACT ACG ACC ACA TCA- 3′) and IS6110R (5′-CCG TAA ACA CCG TAG TTG- 3′), which amplify 106 bp within the mobile genetic element IS6110 specific to the *M. tuberculosis* complex.

### Serum antibody assay

Wells of Immulon™ 2HB 96-well dishes (Thermo Scientific Nunc, Waltham, MA) were coated with 2 µg/ml of *M. tuberculosis* strain H37Rv whole cell lysate (BEI NR-14822) in PBS overnight at 4°C. Excess unbound antigen was removed by washing the wells with 1X KPL wash buffer (KPL, Inc., Milford, MA). The wells were blocked with Blotto (KPL wash buffer containing 5% nonfat dry milk) for 2 hours at room temperature. Serum samples serially-diluted in Blotto were added to the wells. After overnight incubation at 4°C, the wells were washed with 1X KPL buffer, followed by two-hour incubation at room temperature with alkaline phosphatase-labeled goat anti-ferret IgG (KPL). Alkaline phosphatase activity was detected by addition of 100 μl *p*-nitrophenylphosphate (KPL) and measuring the optical density at 405 nm in a Bio-Tek Powerwave XS plate reader (BioTek Instruments, Winooski, VT).

### *In vitro* splenocyte stimulation

Ferret spleens were aseptically harvested and splenocytes enriched by mechanical disruption using a 5-ml syringe and filtration through a 70-μm cell strainer. The cells were pelleted by centrifugation (300 X *g* for five minutes). Erythrocytes were removed by lysing in ammonium chloride-based buffer (ACK Lysis Buffer) for five minutes and splenocytes washed in R10 medium (RPMI 1640 with heat inactivated10% FBS and 2 mM L-glutamine). The cell density was adjusted to 2.5 X 10^7^ cells/ml with freezing medium (10% DMSO in FBS) and cells stored at -80°C in 1.5-ml aliquots. Splenocytes were thawed, seeded into 96-well Costar plates at 1x 10^6^ cells/ml per well, and stimulated at 37°C, 5% CO_2_, for either one or six days with various stimulants such as *M. tuberculosis* strain H37Rv whole cell lysate (BEI NR-14822) or recombinant ESAT-6 (BEI NR-14868). The positive controls were phorbol 12-myristate, 13-acetate (PMA) and ionomycin for day-1 and phytohemagglutinin (PHA) for day-6 samples. At the indicated time points, culture supernatants were collected, filtered through 0.22-µm PVDF membranes, and stored at -80°C until assayed. The IFN-γ levels in the thawed supernatants were measured using an anti-ferret IFN-γ ELISA kit (Mab Tech, Nacka, Sweden).

### Cytokine/chemokine analysis – lung tissue RNA isolation, cDNA synthesis, and quantitative reverse transcription PCR

At necropsy, four mm segments from lung biopsies were collected from various lobes, pooled and, homogenized in five ml Trizol (Sigma-Aldrich, St. Louis MO) or RNAzol RT (Molecular Research Center, Cincinnati OH). Total RNA was extracted from one ml homogenate as per manufacturer’s protocol. Approximately 10 µg of total RNA was treated with RQ1 RNase-free DNase (Promega Corp., Madison WI) and purified using Monarch RNA Cleanup Columns (New England Biolabs, Ipswitch MA). The RNA quantity was assessed by measuring the absorbance ratios of 260/280 and 260/230 nm in an Epoch plate reader (Biotek, Winooski VT). For cDNA synthesis, reverse transcription was performed with LunaScript (New England Biolabs). Each reaction included 1× LunaScript Super reaction mix and one μg of RNA template. This mixture was incubated at 25°C for two minutes, then at 55°C for 30 minutes followed by inactivation at 95°C for one minute. Real-time PCR was performed with Fast SYBR Green Master Mix (Thermo Fisher Scientific, Waltham MA) using a Mx3000P/3005P thermocycler (Stratagene, San Diego CA). Briefly, a 1:10 dilution of the cDNA was added to a PCR reaction consisting of Fast SYBR Green Master Mix and 150 nM of each primer (**Supplemental Table 1**). The real-time PCR cycler program used was 50°C for two minutes, 95°C for 10 minutes, followed by 40 cycles at 95°C for 15 seconds and 58°C for one minute. The dissociation curve was obtained by heating from 60°C to 95°C. The gene-expression levels were normalized to 18S rRNA as an internal control and presented as fold change over uninfected control using the delta-delta Ct method (ΔΔCt).

### Data analysis

Statistical analysis was performed using PRISM software, version 9.0 (GraphPad Software, Inc., San Diego, CA). Statistical significance was set at *p* ≤ 0.05. When more than two groups were compared, one-way analysis of variance (ANOVA) was used with Bonferroni’s *post hoc* analysis or another nonparametric equivalent. Regarding animal numbers, for new outbred models of infectious diseases such as the Syrian hamster and COVID-19 (Chan et al., 2020), 5 or 6 animals per group with n=3 animals per time point were used and assessed. This was the strategy when possible in our study.

## Results

### Infection study

To investigate the ferret as a potential animal model for human tuberculosis, a study was designed to monitor disease status, immune responses, bacterial burdens in organs, and pathology with standard doses of *M. tuberculosis* used in mouse and guinea pig studies. As detailed in the Methods section, this experiment consisted of 18 ferrets (3 groups of 6) infected *via* the intratracheal route with a low (1 – 5 x 10^1^), medium (1 – 5 x 10^2^), or high (5 – 10 x 10^3^) CFU dose of *M. tuberculosis* strain Erdman and an uninfected ferret euthanized at the completion of this experiment. At 4 and 7 weeks post infection, three animals in each group were euthanized and examined. The time course and various parameters examined in this study are outlined in **Supplemental Figure 1** and **Table 1**, and results are presented below.

### Observable clinical signs of tuberculosis

#### Body weight

As described in the Methods section, body weight was assessed as a comparison of weight gain among the various study groups. All infected (low, medium, or high dose) ferrets had less weight gain as compared to the uninfected control, especially towards the later time points. (**Supplemental Figure 2A**).

#### Body temperature

Individual animal temperatures spiked randomly during the course of the study, with the mean change in temperature not being significantly different compared to baseline measurements prior to infection in the infected and non-infected animal groups **(** **Supplemental Figure 2B** **)**. However, the infected ferrets in the high-dose group generally had more weeks with greater temperature increases than the other groups indicating progression to an active disease.

#### Lethargy and respiratory distress

In these initial infection experiments, again, likely due to the limited seven-week duration, only the animals infected with the highest dose showed moderate lethargy and respiratory stress as displayed by decreased movement and occasional coughing.

### Delayed-type hypersensitivity

**Figure 1:**
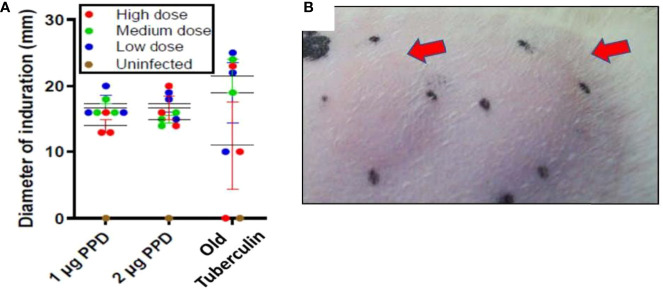
Infection study: Ferrets infected with *M. tuberculosis* strain Erdman generate a strong delayed-type hypersensitivity (DTH) response to PPD. The flanks of the infection-study ferrets were shaved and different areas injected intradermally with 1 or 2 µg PPD or 1,000 IU Old Tuberculin 7 weeks after intratracheal infection with the indicated *M. tuberculosis* dose. The diameter of the induration (mm) was measured at 24 hours after injection. The black dots mark the ends of the induration. **(A)** Data represent mean ± SEM from groups of 3 infected ferrets and 1 uninfected ferret, and **(B)** arrows indicate typical areas of induration observed in ferrets given PPD.

Ferrets, like guinea pigs, cattle, and NHPs, consistently generate a delayed-type hypersensitivity (DTH) immune response to intradermal tuberculin purified protein derivative (PPD) by 4-8 weeks after *M. tuberculosis* infection. In week 7 of the initial infection study, DTH was assessed at 24, 48, and 72 hours post injection with 1 and 2 µg PPD or 1,000 IU Old Tuberculin (OT) in different shaved areas over the flanks. Old Tuberculin is a crude *M. tuberculosis* cell wall preparation used to measure DTH in NHPs. All ferrets at 24 hours post-injection with PPD developed induration irrespective of the intratracheal dose of *M. tuberculosis* received (**Figures 1A**, **B**). The mean diameter of the induration in response to PPD at 24 hours in low-dose *M. tuberculosis*-infected animals was 17 ± 1.2 mm, while medium-dose infection generated a mean diameter of 15 ± 0.5 mm, and high-dose infection produced 16.6 ± 1.7 mm. There were no significant differences between the groups based on the size of induration. The induration in the ferrets peaked between 24 and 48 hours post-PPD administration. The ferrets also generally responded to Old Tuberculin; however, the induration reaction was variable from 0 to 25 mm (**Figure 1A**). There was no induration or erythema observed in the uninfected ferret post intradermal PPD and Old tuberculin administration.

### MGIT culture and IS6110 PCR of non-invasive and post-mortem samples

At 4 and 7 weeks post infection, nasal washes and throat swabs from individual ferrets and fecal samples from the cage pans were collected, decontaminated, and inoculated into MGIT cultures. Positive MGIT samples were further analyzed by real- time PCR using *M. tuberculosis*-specific IS6110 primers for confirmation. As detailed in **Supplemental Tables 2A**, **B**, for each 6-ferret infection-dose group, three animals were sampled at week 4 and three at week 7. Combining results from both time points, two of six nasal wash samples from high-dose and one of six medium- and one of six low-dose samples were positive by MGIT and IS6110 PCR. Throat swabs from five of six ferrets in the high-dose group were MGIT and IS6110 positive, but not for any animals in the other groups. Fecal samples at the week 7 time point were MGIT and IS6110 positive primarily in the high-dose group. Lastly, stomach contents from two of three high-dose and one of three medium-dose ferrets at the week 7 time point were MGIT and IS6110 positive (**Supplemental Tables 2A**, **B**).

**Figure 2:**
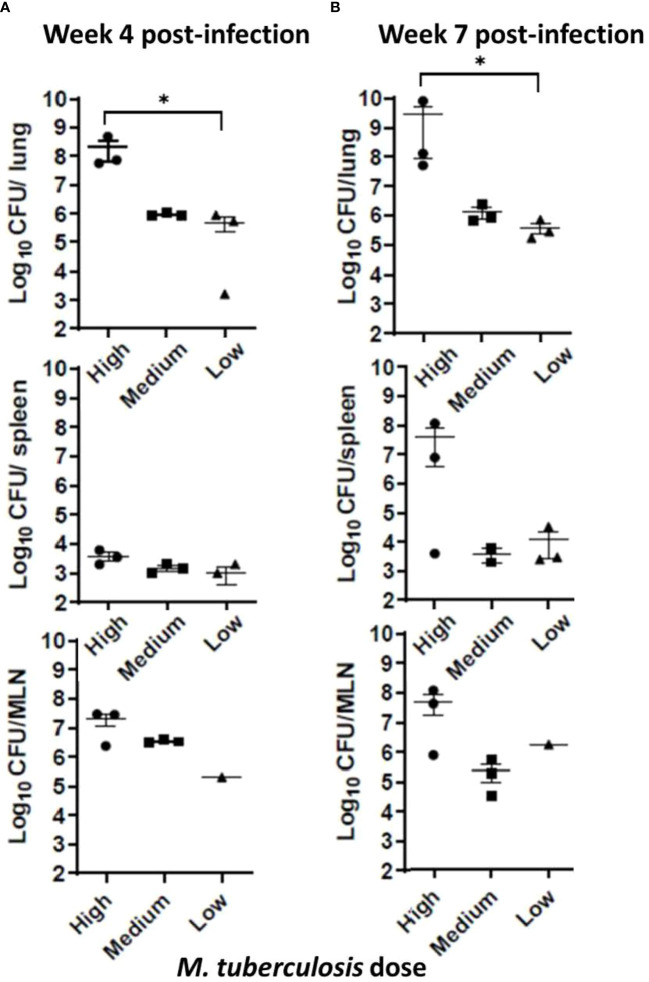
Infection study: Bacterial loads in infected ferret tissues. Mycobacterial CFU in homogenates of infection study ferret lungs (upper row), spleen (middle row), and mediastinal lymph nodes (MLN) (lower row) were obtained at **(A)** 4 weeks and **(B)** 7 weeks post infection with low, medium, or high dose of *M. tuberculosis* bacilli. Data represent mean ± SEM from 3 ferrets in each group at each time point, except the mediastinal lymph node (MLN) from the low dose-infected group (n = 1), since we could not isolate them cleanly during necropsy. Data were analyzed by One-way ANOVA and Kruskal-Wallis comparison test, *p < 0.05.

At the conclusion of the noninvasive sample collections at weeks 4 and 7, sets of animals were euthanized, organs harvested, one-half of each organ homogenized, and dilutions placed in MGIT culture tubes; if positive, the contents were confirmed by 1S6110 PCR. To evaluate *M. tuberculosis* burdens in organs, viable CFU plating was performed using lung, spleen, and lymph node tissues. The *M. tuberculosis* bacilli instilled in the ferret trachea multiplied in the lungs to around 1 x 10^6^ CFU in the low- and medium-dose groups, and to approximately 1 x 10^8^ CFU in the high-dose group by week 4; however, at week 7, only the high-dose group showed increased bacterial counts (**Figures 2A**, **B**). Also, *M. tuberculosis* bacilli could be cultured from the extrapulmonary tissues – spleen, and mediastinal lymph nodes (MLN) indicating dissemination in all of the groups at both 4 and 7 weeks, post infection. Interestingly, bacterial loads in the lungs, spleen, and lymph nodes in the low- and medium-dose groups remained relatively constant between the 4- and 7-week time points indicating possible containment of the infection at least during this study period (**Figure 2B**).

### Post-mortem examination and lesion score

**Figure 3:**
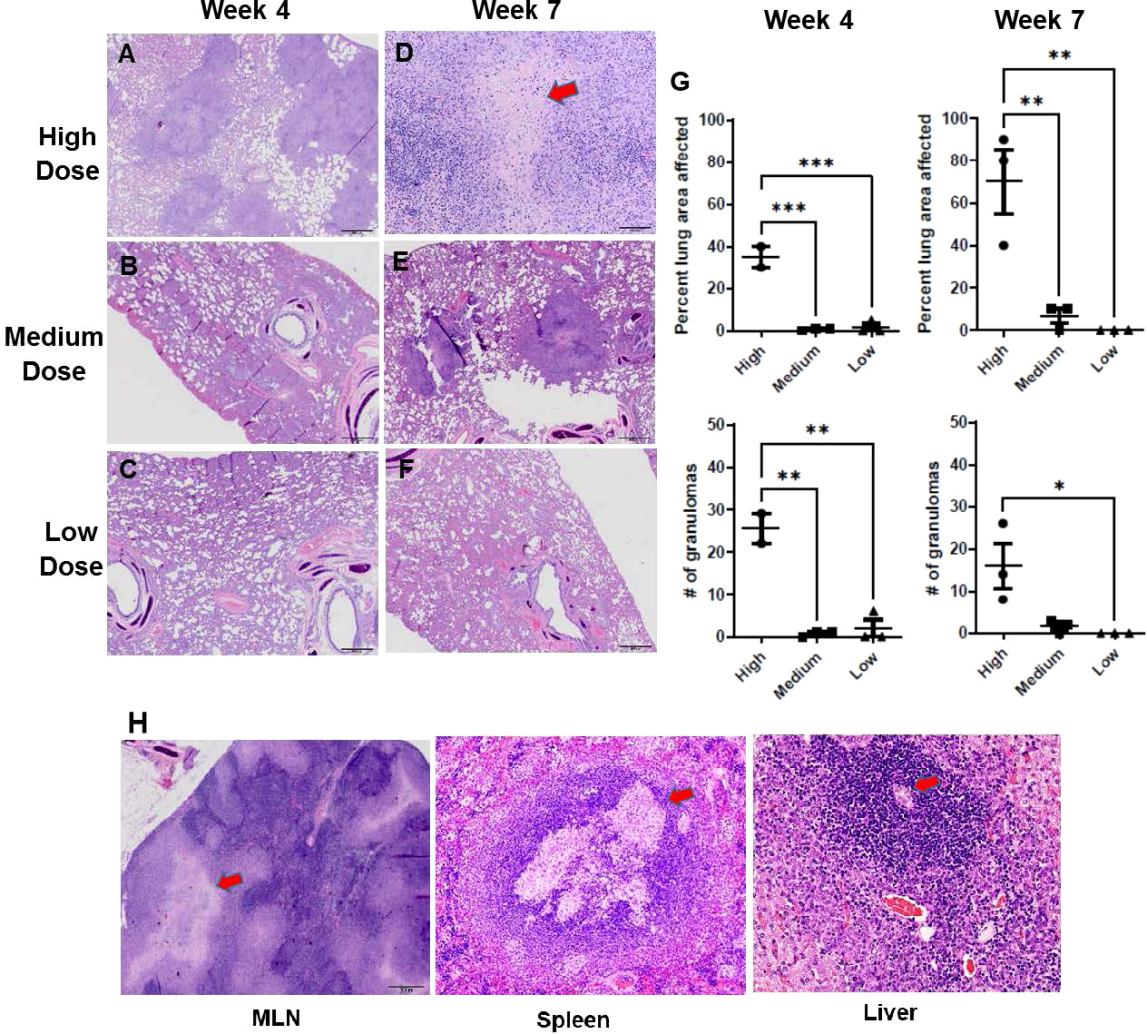
Infection study: Histopathology of the ferret lung post-challenge. Representative hematoxylin and eosin (H&E)-stained lung sections from ferrets 4 weeks **(A-C)** and 7 weeks **(D-F)** after infection with a high, medium, or low dose of *M. tuberculosis* **(G)** Percent of examined lung area affected and number of granulomas per examined lung area 4 weeks and 7 weeks after infection with high, medium, or low-dose *M. tuberculosis*. Data represent n = 3 ferrets for low-and medium-dose infections and n = 2 for the high-dose group. Data analyzed by One-way ANOVA and Bonferroni’s multiple comparison test, *p < 0.05, **p <0.01, ***p < 0.001. **(H)** Representative H&E-stained mediastinal lymph node, spleen, and liver sections from ferrets 7 weeks after infection with high-dose *M. tuberculosis*. 400X magnification. Red arrows on high-dose, week-7 sections **(D, H)** identify caseous necrotic lesions.

With the remaining organ sections collected and examined post mortem, histology was reviewed with a specific focus on granuloma characteristics described in the Methods section. The infected ferrets in the infection study showed lung pathology generally correlating with the infectious dose of *M. tuberculosis* bacilli received and the interval since initial infection (**Figures 3A**-**F**). For example, one of three ferrets that received a high dose of *M. tuberculosis* bacilli developed caseous, necrotic granulomas by week 7 post infection, while two of three ferrets that received a medium dose had granulomas with limited areas of necrosis. The ferrets infected with the low *M. tuberculosis* dose did not generate caseous granulomas in the assessed lung sections within the 7-week study period. There also were significant differences in the numbers of granulomas generated, and thus, the percent lung affected among the various infection groups at the two time points studied correlated with the infection dose. In the high-dose group, the number and distribution of lung lesions increased as the disease progressed from weeks 4 to 7 and was significantly greater than in either the medium- or low-dose groups (**Figure 3G**). The spleen from each of the *M. tuberculosis*-infected ferrets was observed to be enlarged, while the corresponding lymph nodes were palpable and prominent, and the centers of these lymph nodes were caseous. Similarly, it was observed that the livers from the high-dose infection group had white foci visible on their surfaces. Microscopically, increased cellular infiltration in mesenteric lymph nodes (MLN), spleen, and liver was observed by H&E staining (**Figure 3H**).

### Immunological responses

Active tuberculosis in humans is characterized by the generation of cellular and humoral responses to *M. tuberculosis* antigens and high levels of many cytokines and chemokines (Domingo-Gonzalez et al., 2016). The present study assessed antibody responses in the serum to *M. tuberculosis* whole cell lysates and expression of a representative group of cytokine and chemokine genes in lung tissues of infected animals.

#### Cytokines and chemokines

**Figure 4:**
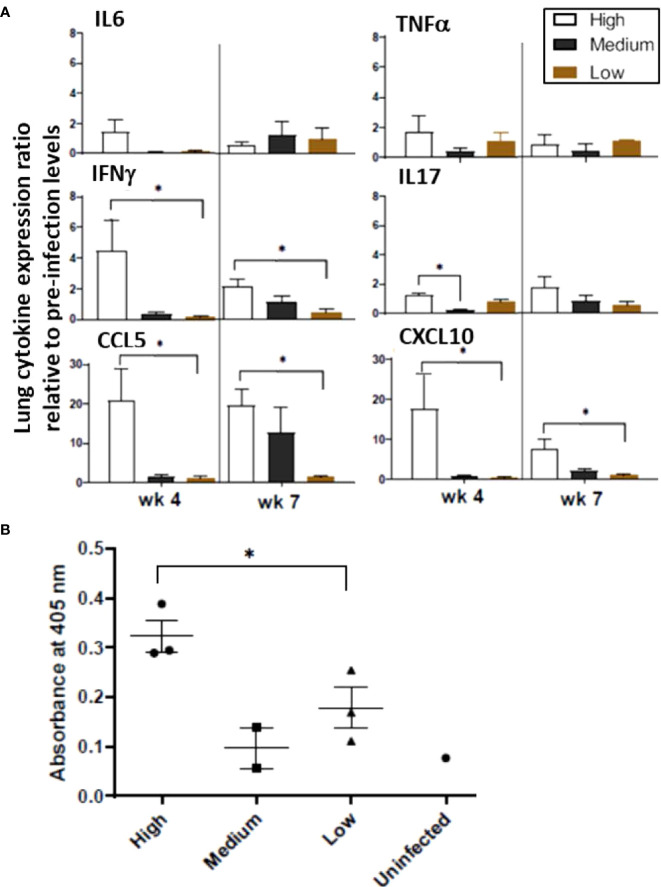
Infection study: Ferret lung cytokine and serum antibody levels. **(A)** Expression of the indicated cytokines/chemokines in the lungs was measured by qRT-PCR. Pooled RNA from lung tissue of infection study ferrets at 4 and 7 weeks post infection with high, medium, or low doses of *M. tuberculosis* was examined. Data represent fold changes relative to pre-infection levels and 18S as house-keeping gene. **(B)** Sera from ferrets at week 2,4 and 7 weeks after intratracheal infection with the various doses of *M. tuberculosis* bacilli were assayed for antibodies against *M. tuberculosis* whole cell lysate by ELISA. The y-axis indicates absorbance at wavelength 405 nm. The x-axis indicates the serum dilution. Serum samples were measured in duplicate. Data represent mean ± SEM from 3 groups at various time points. The groups were analyzed by One-way ANOVA for non-parametric samples followed by Kruskal-Wallis and Dunn’s multiple comparison; *p < 0.05.

Expression of various genes encoding pro-inflammatory cytokines and chemokines was examined in the lung homogenates, including IL-6, IL-17, CCL5 (RANTES), CXCL10 (IP10), TNF-α, and IFN-γ. Each of these genes tended to be expressed at higher levels at the 4-week time point in animals infected with high-dose *M. tuberculosis* than in those that received medium or low doses while at the 7-week time point, transcription levels were more varied (**Figure 4A**). Regarding individual gene expression patterns, IL-17 is important for early defense against bacterial pathogens and induces pro-inflammatory cytokines and chemokines; thus, it is noted that expression of the IL-17 gene increased in the high-dose infection group by 4 weeks post infection and remained elevated at 7 weeks post infection. In the medium-dose animals, the expression levels increased by 7 weeks post infection; however, no change was detected in the low-dose group. CXCL10 is a major biomarker for active tuberculosis and is induced by IFN-γ (Jeong et al., 2015). Expression of the CXCL10 gene in the high-dose group was 16-fold higher than uninfected controls at 4 weeks post infection and remained elevated (~7-fold) at 7 weeks post infection; but the expression levels in the medium- and low-dosed ferrets remained low. Concomitantly, expression of the gene encoding IFN-γ was elevated four-fold in the high-dose ferrets by week 4 and dropped to two-fold by week 7 post infection, but levels remained low in the other groups. In addition to CXCL10 and IFN-γ, the mRNA levels of the CCL5 gene also increased in the high-dose group at 4 weeks post-infection (20-fold) and remained at the same level at 7 weeks. The CCL5 mRNA levels increased in the medium-dose group by 7 weeks post-infection, but there was no change in the low-dose group. At 4 weeks post-infection, TNF-α gene transcription was elevated approximately 1.6-fold in the high-dose group with lower levels detected in the medium- and low-dose groups, while at 7 weeks, levels were low in all groups. Together, these data suggest that ferrets infected with a high dose of *M. tuberculosis* mount stronger pro-inflammatory responses in the lungs in the form of elevated expression of the genes for IL-6, IL-17, CXCL10, IFN-γ, and CCL5, as compared to the medium and low-dose infection groups. With the exception of CCL5, the inflammatory responses in the high dose group were usually higher 4 weeks post infection and then they declined by 7 weeks post infection.

#### Humoral

The IgG response against *M. tuberculosis* whole cell lysate in the infected ferrets was generally low at week 7 post infection. However, the animals infected with a high *M. tuberculosis* dose generated the higher antibody responses by week 7 post infection (**Figure 4B**). The role of humoral immune responses in controlling *M. tuberculosis* infections is still under investigation. The response is heterogenous and depends on the stage of disease (Demkow et al., 2007). Poor antibody response to an intracellular pathogen is not surprising. Moreover, higher IgG antibody levels correlate with advanced forms of the tuberculosis disease. Hence, lower IgG responses against *M. tuberculosis* cell lysates in sera from low and medium-dose infected ferrets indicate these lower doses animals may be able to resolve the infection. A longer-term study with low and medium-dose infections will be performed to confirm this hypothesis.

### Transmission study – medium-high dose

A direct transmission study was performed according to the outline in **Supplemental Figure 1**. As indicated in **Table 1**, routine monitoring with non-invasive tests included TST and analysis of nasal washes, throat swabs, and feces by MGIT culture and IS6110 PCR. Most ferrets were euthanized 27 weeks after the initial infection of the transmitter ferrets, at which time necropsy specimens were collected and analyzed. In this study, animal-to-animal transmission was observed along with disease symptomologies and interestingly, transmission was most effective when sentinel ferret, SE5 was exposed to a partnering transmitter ferret TR4 which was presenting with acute disease rather than when exposed to a transmitter ferret only 24 hours after infection. Data are summarized (**Supplemental Table 3A** **)** and relevance is discussed in more detail below.

### Observable clinical signs of tuberculosis

#### Weight loss

As described in the Methods section, eventual weight loss in both TRs and SEs was hypothesized and observed over the course of the 27-week experiment (**Supplemental Figure 3A**). Interestingly, weight loss in the TR and SE pairs was comparable. TR1 and its cage mate SE1 lost 14% and 11%, and TR2 and SE2 lost 10% and 15%, respectively, of their maximal body weight by week 27. In the same period, TR3 and SE3 lost 25% of maximal body weight. SE5, SE6, and SE7 had the lowest weight losses likely because exposure time with their respective TRs was only 4 weeks.

#### Body temperature

Body temperatures spiked at some time points in the TR animals, but was more often observed at +1°C levels in SE1, SE3, SE4 ferrets over the course of the study (**Supplemental Figure 3B**). Spiking fever is a characteristic of human tuberculosis (Behr et al., 2018); however, it is interesting to note that it was observed to a greater extent in the SE animals.

#### Lethargy and respiratory distress

In general, TR and some SE animals all showed modest lethargy during the course of this study compared with the uninfected controls.

### Delayed-type hypersensitivity

**Figure 5:**
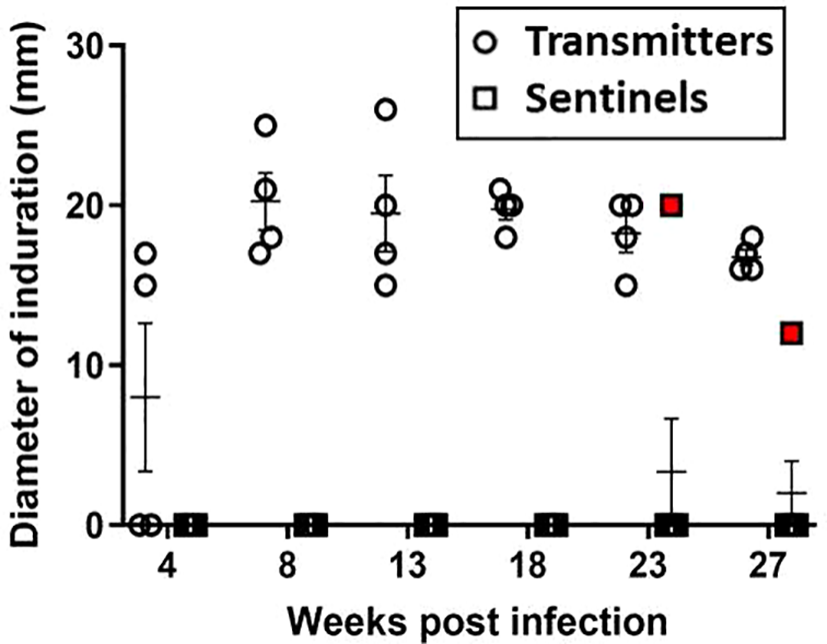
Transmission study using medium-high dose-infected transmitters: DTH reaction to PPD. Sustained DTH to 2 µg PPD in *M. tuberculosis*-infected transmitter (TR) ferrets was observed by week 8 post infection. One sentinel, SE5 (red boxes) co-housed with TR4 in week 17 of the study, tested positive beginning 5 weeks later. Data represent mean ± SEM of n = 4 transmitters and n = 7 sentinels.

Skin test responses were performed by intradermal injection of 2 μg PPD and induration measured after 24 - 48 hours. At 8 weeks post-infection, all TRs were PPD-positive with induration (**Figure 5**); however, none of the original surviving SEs (SE1, SE2, and SE3) co-housed with TRs 24 hours post-infection converted their skin tests. Interestingly, these sentinels remained TST-negative but had positive *M. tuberculosis* CFU in nasal washes; this has also been reported in humans exposed to infected patients (Jones-López et al., 2015). Equally interesting, ferret SE5 developed a positive TST 6 weeks after exposure to its infected cage-mate, TR4; recall that TR4 had been infected 17 weeks earlier, and SE5 was substituted for the original sentinel animal, SE4. Sentinel animals SE6 and SE7, which were substituted for SE1 and SE2, respectively, at 23 weeks post-infection, remained TST negative until study termination 4 weeks later; this was not unexpected given the short exposure duration to their respective TRs. The mean diameter of induration in TRs at study termination was 18 mm (range 15 – 26 mm). The control ferrets did not develop immune responses to PPD over the course of the study.

### MGIT culture and IS6110 PCR of non-invasive and post-mortem samples

**Figure 6:**
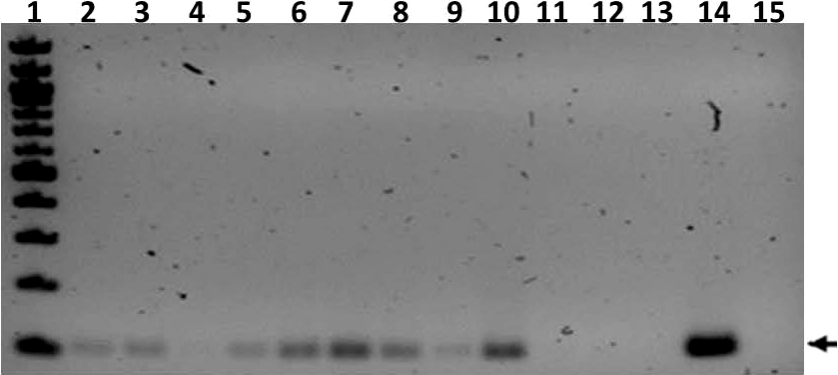
Transmission study using medium-high dose-infected transmitters: IS6110 PCR of ferret nasal wash samples. Shown is a gel image of IS6110 PCR products from transmitter (TR), sentinel (SE), and uninfected control (UI) ferrets resolved on a 2% agarose gel. The arrow indicates the expected band size corresponding to the 106 bp IS6110 amplicon. Lane 1 = 100 bp ladder (NEB), 2 = TR1, 3 = TR2, 4 = TR3, 5 = Tr4, 6 = SE1, 7 = SE2, 8 =SE3, 9 = SE5, 10 = SE6, 11 = SE7, 12 = UI (F8), 13 = UI (F9), 14 = positive control amplified with *M. tuberculosis* genomic DNA template, and 15 = negative control DNA. The samples were collected at study termination.

PCR of nasal wash samples collected at week 27 post infection resulted in IS6110 positivity for all ferrets except for TR3 and SE7, and the uninfected controls (**Figure 6**). Nasal washes from TR1, SE1, and SE2 were also *M. tuberculosis* culture positive. Broncho-alveolar lavages were MGIT and/or culture positive for all transmitter animals and all but two of the sentinels (**Supplemental Table 3A**).

**Figure 7:**
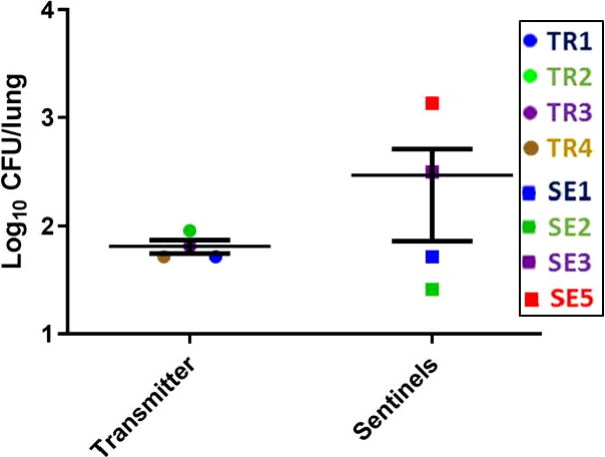
Transmission study using medium-high dose infected transmitters: Mycobacterial loads in ferret lungs. At the termination of the transmission study (week 27 post-infection), lung homogenates of the transmitter and sentinel ferrets were processed for *M. tuberculosis* CFU. Data represent mean ± SEM; n = 4 transmitters and n = 7 sentinels of which only 4 had measurable *M. tuberculosis* CFU detected.

The lung CFU burdens in the TR ferrets averaged 1 x 10^2^ CFU at week 27 post infection (**Figure 7**), while the lung CFU in the SEs was variable, likely indicating more recent and/or ongoing infections. The lung and spleen homogenates from all TR and SE ferrets in this transmission study were also positive by MGIT culture and IS6110 PCR, except for the TR2 lung, which was only culture positive, and the TR4 spleen, which was negative for both tests (**Supplemental Figure 4**, **Supplemental Table 3A**).

### Post-mortem examination and lesion score

**Figure 8:**
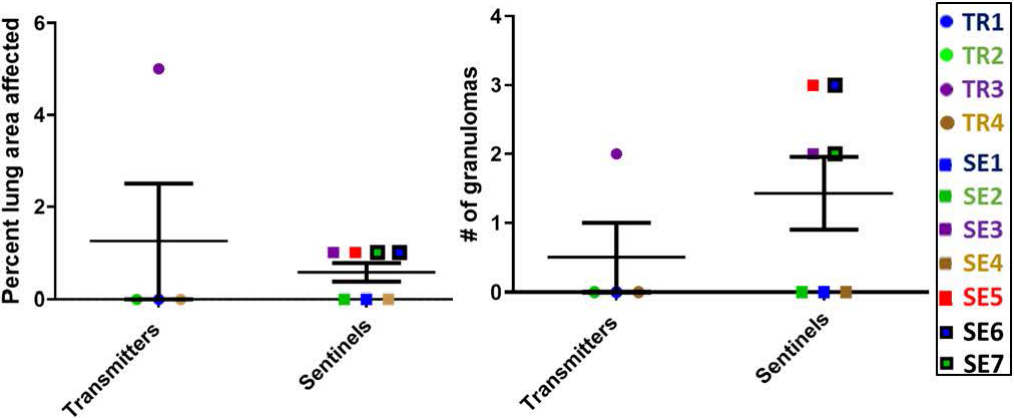
Transmission study using medium-high dose infected transmitters: Histopathology of direct-contact transmission study ferret lungs. Percent of examined lung area affected 27 weeks post-TR infection and numbers of granulomas per examined lung area are shown. Data represent mean ± SEM from groups of 4 ferret transmitters and 7 sentinels.

At the completion of this transmission study, the numbers of granulomas in transmitter and sentinel ferrets varied overall, but there were interesting observations. For example, of the four transmitter ferrets, only TR3 had lung granulomas by 27 weeks post-infection; however, SEs co-housed with TRs at an advanced stage of disease for a few weeks (SE5, SE6 and SE7) along with one sentinel co-housed for the entire 27-week period (SE3) developed granulomas of varying sizes (**Figure 8**). Uninfected, negative-control ferrets had no granulomas.

### Immunological responses

#### Cytokines

**Figure 9:**
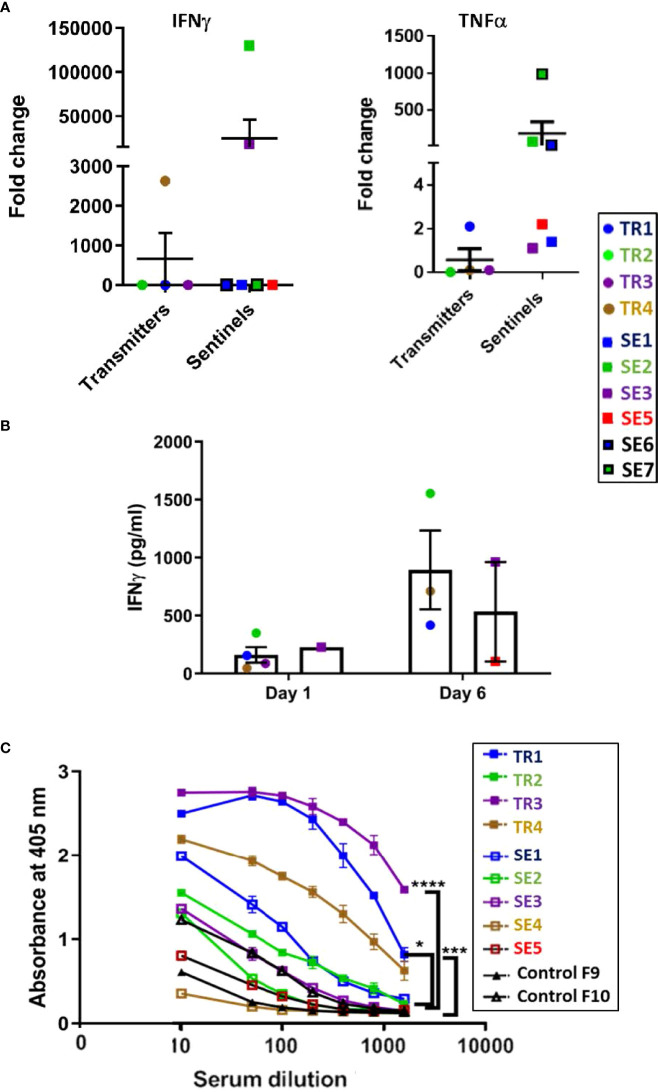
Transmission study using medium-high dose-infected transmitters: Ferret lung cytokine gene expression and splenocyte and antibody responses to M. tuberculosis antigens. **(A)** Expression of genes encoding IFN-g and TNF-a was measured by qRT-PCR of RNA extracted from lung homogenates of uninfected controls and transmitter and sentinel ferrets 27 weeks after initial transmitter infection. Data represent mean ± SEM of gene expression change in four transmitter or six sentinel ferrets relative to uninfected controls. Animals not represented in figure did not have gene expression levels greater than controls. SE4 lung homogenates were not assessed in this experiment. **(B)** Splenocytes harvested and cryopreserved from transmitter and sentinel animals were thawed, washed, and 2X106 cells were cultured in duplicate with *M. tuberculosis* whole cell lysate (WCL). Culture supernatants were assayed at day 1 and day 6 post stimulation for IFN-g release by ELISA. Positive controls were stimulated with PHA or PMA + ionomycin. Cells in medium only served as the negative control. Statistical analysis was performed using a Mann-Whitney test for four transmitter or six sentinel ferrets relative to uninfected controls. SE4 splenocytes were not assessed in this experiment. **(C)** Sera were collected from uninfected controls, and transmitter and sentinel ferrets 27 weeks after initial transmitter infection and were assayed for antibodies against M. tuberculosis whole cell lysate by ELISA. The y-axis indicates absorbance at a wavelength of 405 nm. The x-axis indicates the serum dilution. Serum samples were diluted and measured in duplicate. No antibodies were detected in SE6 and SE7. Data analyzed by One-way ANOVA and Bonferroni’s multiple comparison test, *p < 0.05, ***p < 0.001, ****p < 0.0001.

At the euthanasia endpoint, only TR4 expressed elevated transcription of the IFN-γ gene compared to the other transmitter animals, while all except TR1 expressed only background levels of TNF-α mRNA (**Figure 9A**), confirming the observations from the infection experiment that ferrets infected with a moderate dose of *M. tuberculosis* express a generally low level of pro-inflammatory cytokines. Interestingly, the IFN-γ levels of SE2 and SE3, as well as TNF-α levels in SE2, SE6 and SE7, were high compared to SE1, SE3, and SE5. High TNF-α in SE6 and SE7 indicate that within 4 weeks of being housed with transmitters TR1 and TR2, respectively, the sentinels produce TNF-α to contain the infection. It is well known that infected macrophages are the main source of TNF-α in the lungs and act as first line of defense.

Cultured ferret splenocytes were assessed for IFN-γ expression *via* ELISA when stimulated with *M. tuberculosis* whole cell lysates. It was observed that 3 out of 4 TRs, and 2 of 6 SEs released IFN-γ upon stimulation with *M. tuberculosis* whole cell lysate by assay day 6 (**Figure 9B**).

#### Humoral

All four transmitters developed strong anti–*M. tuberculosis* whole cell lysate serum IgG titers by 27 weeks post infection (**Figure 9C**). Co-housed sentinels SE1, SE2, and SE3 also developed anti-*M. tuberculosis* antibodies. However, one of the two uninfected control ferrets had a low titer of anti-*M. tuberculosis* IgG that was higher than sentinel ferret SE5 at the time of euthanasia. This was a specific reaction since the pre-immune sera from this control ferret did not show a detectable titer. No other tests were positive for either control (**Supplemental Table 3A**). The control ferrets were housed in open cages in a different animal facility outside of biocontainment. It is possible that the observed minor response in this ferret may have been due to exposure to environmental mycobacteria.

### Transmission Study – very-high dose

To determine if transmission of *M. tuberculosis* between ferrets is more efficient using a higher infection dose in the transmitter animals, six transmitter animals (TR-A1 – TR-A6) were infected *via* intratracheal instillation with 5 x 10^4^ CFU *M. tuberculosis* bacilli and each placed in separate aerosol-transmission cages with perforated stainless steel dividers and unidirectional airflow from the upstream to downstream sides of the cages. Five weeks after the transmitter ferrets were inoculated, direct sentinel ferrets SE-D1, SE-D2, and SE-D3 were co-housed with TR-A1, TR-A2, and TR-A3, respectively, on the upstream side. Six aerosol sentinel ferrets (SE-A1 – SE-A6) were placed on the downstream side of the cages from transmitters TR-A1 – TR-A6, respectively. Also at week 5 post infection, TR-A4 and TR-A5 reached humane end points and had to be euthanized. Because of the severity of the weight loss by all transmitters, the remaining transmitters were placed on Oxbow critical care diet and were observed closely and weighed twice daily for the duration of the study. All sentinels remained in their respective cages. At week 8 post infection, TR-A3 and TR-A6 also reached their humane endpoints and had to be euthanized; tuberculin skin tests on the remaining transmitters (TR-A1 and TR-A2) were performed on week 9 post-infection, and on all sentinels on week 4 of co-housing (corresponding to TR-A1 and TR-A2 week 9 post-infection). Nasal washes collected at these same time points were used for IS6110 PCR. At week 10 post transmitter infection, the study was terminated and tissues collected from all remaining animals. Swabs of the exhaust plenums from the cages holding TR-A1 and TR-A2 (cages 1 and 2), the final two remaining transmitter animals, were taken within 24 hours of study termination and found to be IS6110 PCR positive. This result independently confirmed directional transmission of *M. tuberculosis* bacilli from the upstream to downstream sides of the cages (**Supplemental Figure 5**). All data are summarized in **Supplemental Table 3B**, and relevance is discussed in more detail below.

### Observable clinical signs of tuberculosis

#### Weight loss

Weight loss in all the transmitter ferrets over the course of the 10-week experiment was evident with losses ranging from 18-35% (**Supplemental Figure 6A**). Interestingly, aerosol-only sentinels, SE-A2, SE-A3, and SE-A5, and direct-contact sentinel, SE-D2, also lost 7%, 12%, 10%, and 16%, respectively, of their starting weights (**Supplemental Figure 6B**).

#### Body temperature

A spike in temperature was observed in all transmitters within a week of infection, and temperatures remained mostly elevated throughout the course of the study (**Supplemental Figure 6C**). Interestingly, the temperature of the transmitter TR-A4 dropped significantly on the day it reached humane endpoint. The temperature change in the sentinels was more sporadic (**Supplemental Figure 6D**).

#### Lethargy and respiratory distress

All transmitter and direct contact sentinel ferrets showed significant lethargy and acute coughing as time progressed compared with uninfected controls.

### Delayed-type hypersensitivity

Tuberculin skin testing (TST) was performed on transmitter ferrets at weeks 4 and 8 post-infection with 2 μg PPD, and induration was measured at 48 hours. Interestingly, TR-A1 and TR-A2 (animals with the longest infection period in this experiment) developed induration of 12 mm (**Supplemental Table 3B**) while all other transmitters and sentinels were TST negative, even though they were acutely infected and symptomatic. The control ferrets did not develop immune responses to PPD during the course of the study.

### IS6110 PCR of nasal wash

PCR results for nasal wash samples from this transmission study were examined (**Supplemental Figure 7**). Samples collected at week 4 post infection from four transmitters (TR-A2, TR-A4, TR-A5 and TR-A6) were IS6110 positive. The nasal washes collected from all direct-contact sentinels (SE-D1, SE-D2 and SE-D3) as well as one aerosol sentinel (SE-A5) at week 4 of co-housing with transmitters were IS6110 positive (**Supplemental Table 3B**).

### Post-mortem examination and organ viable counts

**Figure 10:**
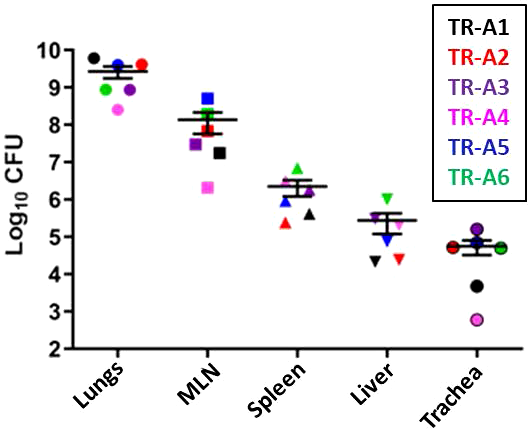
Transmission study using very-high dose-infected transmitters: Bacterial loads in transmitter ferret tissues. Mycobacterial CFU in homogenates of the lungs, mediastinal lymph nodes (MLN), spleen, liver, and trachea were obtained from transmitter ferrets after euthanasia. Data represent mean ± SEM from 6 transmitter ferrets.

The disease outcomes, including weight loss (**Supplemental Figures 6A**, **B**) and breathing abnormalities, of all of the transmitters correlated positively with the bacterial burden in various tissues: lungs, mediastinal lymph nodes, spleen, liver, and trachea (**Figure 10**). Burdens were highest in the lungs, followed by the MLN, spleen, liver, and trachea. All sentinel tissues were plated but no colonies were detected (**Supplemental Table 3B**).

### Immune responses

#### Cytokines and chemokines

**Figure 11:**
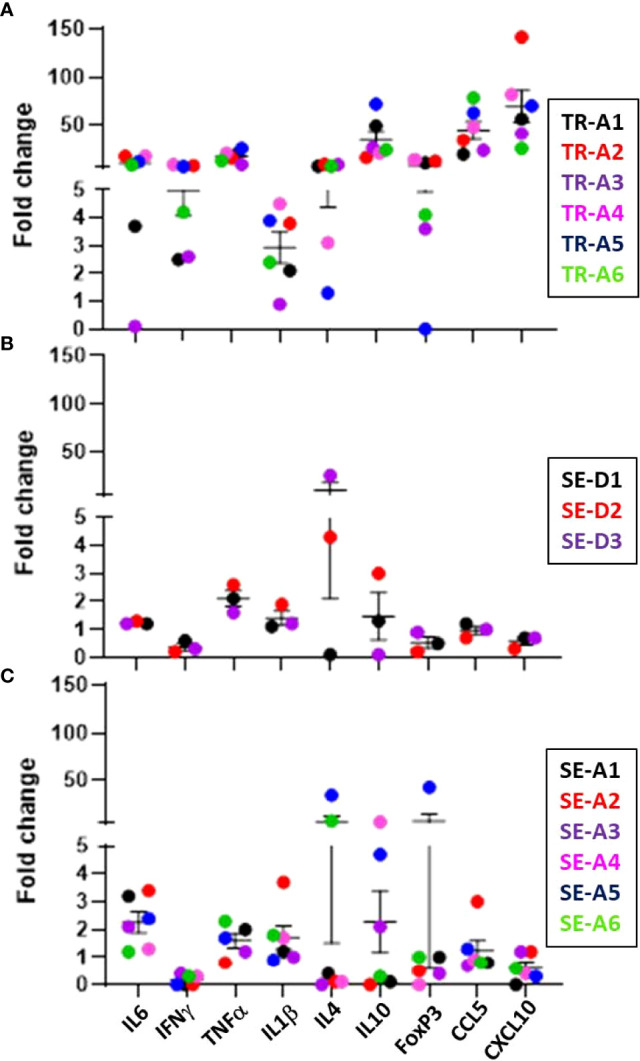
Transmission study using very-high dose-infected transmitters: ferret lung cytokine and chemokine levels. Expression of the indicated cytokine genes was measured by qRT-PCR of pooled RNA from lung tissue from transmitter ferrets at the time of humane endpoint or at study termination 10 weeks post infection. Data represent mean ± SEM of gene expression changes in ferrets relative to n = 3 uninfected control tissues. **(A)** n = 6 transmitters (TRA1- TR-A6), **(B)** n = 3 direct-sentinels (SE-D1 -SE-D3), and **(C)** n = 6 aerosol-sentinels (SE-A1-SE-A6).

Expression of cytokine and chemokine genes encoding CCL5, CXCL10, IFN-γ, TNF-α, IL-1β, and IL-6 in the lungs of infected transmitter, aerosol- and direct-sentinel animals relative to uninfected controls was performed (**Figure 11**). Expression of anti-inflammatory IL-4 and IL-10, and regulatory T cell FoxP3 gene expression was also assessed. All infected transmitters had increased expression of the genes encoding CCL5, CXCL10, and pro-inflammatory cytokines, IL-6 and TNF-α, and IFN-γ. All of the aerosol and direct sentinels had increased IL-6, TNF-α and IL-1β cytokine gene transcription in their lung tissues within 4-5 weeks of exposure to the transmitters. The IFNγ expression in the lungs of aerosol and direct sentinels was negligible. Chemokine CCL5 was upregulated in all sentinels; CCL5 is detected in tuberculosis patients and important in the initial immune response after exposure to *M. tuberculosis* (Vesosky et al., 2010). The anti-inflammatory cytokines IL-4 and IL-10 were high in all transmitters. Interestingly, TR-A4 and TR-A5 that had to be euthanized within 4 weeks of infections, had comparatively lower levels of these anti-inflammatory cytokines than the other transmitters in this experiment. Direct sentinels SE-D2 and SE-D3 expressed high IL-4 levels in lung cells while SE-D1 expressed negligible lung levels. For the aerosol sentinels, only SE-A5 and SE-A6 expressed IL-4 in the lungs. Sentinels, SE-D1 and SE-D2 had high IL-10 levels while SE-D3 expressed negligible levels. Aerosol sentinels SE-A3, SE-A4, and SE-A5 expressed high levels of IL-10, FoxP3 levels in SE-A5 were 40-fold higher than basal levels, and SE-A5 that was co-housed with TR-A2 expressed high levels of IL-4, IL-10 and FoxP3.

#### Humoral

**Figure 12:**
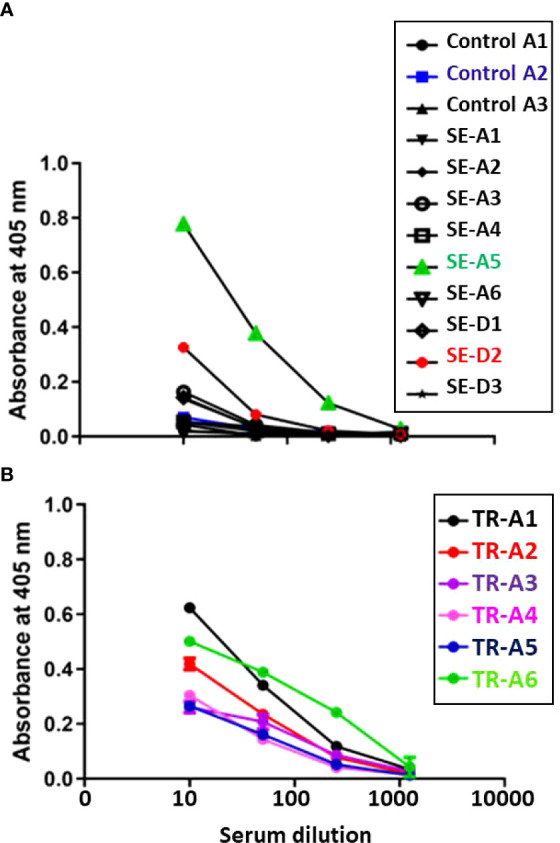
Transmission study using very-high dose-infected transmitters: Humoral antibody response against *M. tuberculosis* whole cell lysate. Sera were collected from **(A)** uninfected control and sentinel ferrets, and **(B)** transmitter ferrets at time of euthannasia. Data represent mean ± SEM of antibody titers against *M. tubercculosis* whole cell lysate by ELISA. The y-axis indicates absorbance at a wavelength of 405 nm. The x-axis indicates the serum dilution. Serum samples were measured in duplicate.

Aerosol sentinel SE-A5 as well as direct sentinel SE-D2 had higher anti- *M. tuberculosis* whole cell lysate IgG levels at week 4-5 post infection, and as expected, all six of the transmitters also developed high antibody titers (**Figure 12**).

## Discussion

The aims of this study were to evaluate infection of ferrets with *M. tuberculosis* and to define an intratracheal infection dose that reproducibly generates disease. In addition, as with influenza virus, we sought to determine if the ferret model could be used as a platform to assess animal-to-animal transmission parameters with *M. tuberculosis*. The data obtained demonstrate that when given a sufficiently-high intratracheal dose of *M. tuberculosis* bacilli, ferrets present with human-like acute disease, and importantly, transmission of bacilli can occur from these experimentally-infected “transmitter” (TR) animals to co-housed naïve “sentinels” (SE).

In the initial infection experiments, we delivered three different infection dose ranges of *M. tuberculosis* bacilli (low: 1 - 5 x 10^1^, medium: 1 – 5 x 10^2^, or high: 5 – 10 x 10^3^ CFU) with up to a 1,000-fold difference between the lowest and highest amounts. We chose to use intratracheal delivery of *M. tuberculosis* bacilli over other methodologies including intranasal or aerosol because we wanted to infect with an exact dose of bacilli (Morales-Nebreda et al., 2014; Ding and Hurt, 2016). The other methods will be assessed in the future, but we did demonstrate in these experiments that intratracheal infection is effective. Overall, clinical signs and pathology correlated with intratracheal dose, with the largest doses (>5 x 10^3^ CFU) generating the most consistent disease signs and lesions. There is an early exponential phase of bacterial replication in the lungs, which disseminates to spleen, lymph nodes, and minimally to the liver between weeks 4 and 7 post infection; this is similar to the CFU plateau in the lungs of mice after 3 weeks. In addition, ferrets that received high *M. tuberculosis* doses were more likely to develop caseous necrotic granulomas by week 7 post infection than those that received a lower dose. At post-mortem examination, a number of tissues appeared to be tuberculous and were assigned a post-mortem score. Differences in lesion score between the lowest and highest infection groups at both 4 and 7 weeks post-infection were statistically significant.

In the transmission studies, transmitter ferrets received either 1 x 10^3^ CFU, a medium-high dose, or 5 x 10^4^ CFU, a very-high dose. By the 27-week termination point of the experiment employing the medium-high dose, all 4 transmitters showed lower bacterial loads in the lungs than the infecting dose; this could be an example of clearance or latent infection. Despite the decreased lung loads, all of these transmitters lost weight over time, had positive TST responses, with one ferret developing necrotic granulomas and losing 25% of maximal body weight. The sentinels in this experiment showed clinical signs of infection with weight loss and *M. tuberculosis* bacilli detected in organs by culture or PCR at necropsy. However, except for SE5, the TST responses were negative; the relevance of this observation is discussed more below. With the transmitters receiving the very-high dose, two of six reached their humane end points and were euthanized 5 weeks after infection, and two more were euthanized at 8 weeks. The study had to be discontinued at 10 weeks due to >30% weight loss, severe lethargy, and coughing in the remaining transmitters. However, as demonstrated by *M. tuberculosis*-positive PCR from nasal washes and lung and spleen sections, the infections in these sentinels were generally more severe than sentinels from the medium-high dose transmitter experiment. Thus, these data demonstrate significant transmission of *M. tuberculosis* bacterial numbers to all direct-contact sentinels, but more robust transmission occurred, not surprisingly from the transmitters given higher infection doses.

An additional component of interest related to the very-high infectious dose transmitter experiment is that the ferrets employed had been previously infected and cleared of H1N1 influenza virus strain CA/09 prior to being included in our study. CA/09 is known to be of low virulence in ferrets (Han et al., 2019), and all animals in our study were shown to be clear of virus 8-12 weeks prior to *M. tuberculosis* infection. Humans infected with influenza virus, and then infected with *M. tuberculosis* 5-7 days later have been shown to develop significantly enhanced tuberculosis disease (Walaza et al., 2015). It is hypothesized that this enhanced mycobacterial replication in the lungs with increased disease manifestations is due to elevation in type I interferons generated by the viral infection (Redford et al., 2014). How long after the virus is cleared does severe disease symptomology and *M. tuberculosis* bacterial replication in the lungs continue to increase is unknown, but we are very interested in defining how much of the enhanced virulence we observed in our transmitter animals in this study may have been due to the very-high infection dose of *M. tuberculosis* bacilli used versus residual effects from the previously cleared influenza infection. Thus, studies in ferrets employing multiple mycobacterial infection doses and post-virus infection time points are currently being assessed.

The argument can be made that the higher transmitter doses are not physiologically appropriate; however, unlike rodents, ferrets appear to require the higher dose of bacilli to reproducibly generate acute disease, at least when infected *via* the intratracheal route. Animals administered lower doses *via* this route generally appear able to control the infection based on decreasing organ CFU burdens and decreasing numbers of granulomas and percent lung involvement. Thus, a very-high dose (5 X 10^4^ CFU) appears to reproducibly generate severe acute tuberculosis symptomology and pathology that is not controlled. In addition, an exciting possibility that was mentioned earlier, and one that will be examined in subsequent studies, is that some of the lower-dosed animals that showed decreased numbers of viable mycobacteria in the lungs, but remained TST positive and had visible lung granulomas at necropsy, may have established latent infection. This phenomenon has been detected in a closely-related species, the badger, infected with *M. bovis* (Corner et al., 2012). Concomitantly, in human tuberculosis household-contact studies, a minority of exposed individuals develop active disease, while others convert their TST without additional signs or symptoms indicating possible latent infection. In our medium-high dose and very-high dose transmission experiments, the transmitter and sentinel animals closely interacted, and, thus, all exposure methods were employed including *via* inhalation and ingestion of *M. tuberculosis* bacilli. Of particular note, and as previously described in the medium-high dose transmission experiment, the naive sentinel ferret, SE5, introduced into the cage with TR4 at week 17 post-infection converted to TST positive within 6 weeks, with culture- and PCR-positive lung and spleen homogenates detected at necropsy indicating active disease. The other sentinels placed with the transmitters immediately after infection remained negative by TST, but positive by IS6110 PCR and lung culture. Because viable bacilli are detected in the lung alveoli and most of the upper airways from these sentinels but no positive TST was observed, perhaps this particular phenomenon is an early stage of latent infection. These results would support the hypothesis that high doses of *M. tuberculosis* bacteria may be responsible for generating active disease, while continual repeat exposure to low doses may foster latent infection, perhaps due to adaptation by the innate immune response. Perhaps the bacilli are not completely cleared but rather are eventually contained within larger granulomatous structures. This process will be more thoroughly assessed in an upcoming study. Thus, in addition to serving as an active-disease tuberculosis model, the ferret may have potential as a small animal latent tuberculosis model.

In both the artificially- and naturally-infected ferrets in these studies, organs that consistently had the higher bacterial counts included the lungs and mediastinal lymph nodes, with lower bacterial counts commonly observed in the liver and spleen. This is a feature of respiratory infection and confirms delivery of the bacilli to the lower lung where the infection is established in humans (Krishnan et al., 2010; Garcia-Pelayo et al., 2015). However, irrespective of bacterial dose or whether the infection was artificial or natural, several ferrets were found to have *M. tuberculosis* bacilli in the mediastinal lymph nodes and/or in their feces. McCallan et al. (2011) and Gallagher and Clifton-Hadley (2000) reported similar results in ferrets and badgers, respectively, in the mediastinal lymph nodes and feces after aerosol-infection with *M. bovis*. In those experiments, exposure initiated by inhalation of *M. bovis* bacilli can establish infection in the lungs with subsequent spread by hematogenous dissemination to distal lymph nodes and visceral organs (McCallan et al., 2011). In our studies, there was a considerable range in the numbers of bacteria recovered from the lungs and associated lymph nodes versus the visceral organs. Such disparity in the distribution of detected mycobacteria in infected non-human primates has been reported (Lin et al., 2009), which provides a level of validation of the ferret model for mimicking human disease. Tuberculosis in humans caused by the ingestion of milk containing *M. bovis* or *M. tuberculosis* is regularly observed (Srivastava et al., 2008; Khan et al., 2019). Mycobacteria are relatively acid-resistant, and thus, surviving transit through the stomach with ultimate deposition in the intestinal tract and feces is well known (Lin et al., 2009). What is less clear is the precise mechanism by which *M. tuberculosis* (or *M. bovis*) exit the gut to become primarily extrapulmonary or pulmonary infections. In a future study, we plan to compare infection of ferrets *via* inhalation and ingestion routes to assess differences in bacterial trafficking and impact on natural transmission to better understand these pathogens and disease processes.

Influenza virus studies have shown that when infected, ferrets present a similar range of clinical signs as humans, such as fever, lethargy, nasal discharge, sneezing, and coughing. Several studies have shown that the virus has been detected in exhaled air during tidal breathing, as well as during episodes of sneezing and coughing (Fabian et al., 2008). During normal breathing, infectious virus is shed into the air in droplets less than 5 microns, a size that can reach the upper and distal airways (Roberts et al., 2012). In both the infection and transmission studies, coughing by ferrets infected with a high or very-high dose of *M. tuberculosis* was observed within a month of infection. Although measurements were not taken in these experiments to demonstrate the presence of mycobacteria in the resulting droplets or the tidal breathing, an inference can be made that, because viable bacilli were routinely present in the nasal washes and throat swabs and in the exhaust plenums from the cages holding the final two transmitter animals from the very-high dose transmission study, the bacilli were in appropriate locations in the transmitter animals to be expelled during coughing episodes.

It is also known that during infection, rupture of a lung granuloma into the airways can lead to transmission of mycobacteria to other individuals *via* aerosolized droplets (Guirado and Schlesinger, 2013). In our transmission studies, not all transmitter and sentinel animals yielded cultivable colonies at every nasal wash collection point; however, transmitters with more severe disease, such as TR3, TR-A1 and TR-A3 did consistently generate *M. tuberculosis-*positive colonies from their nasal washes and developed necrotic lung granulomas. Going forward, we are confident in the design of the completed transmission experiments; however, we would like to more thoroughly assess aerosol-only transmission. To do this, more lengthy exposure times, and inclusion of more than one transmitter animal on the transmission side of the unidirectional airflow special cages will be required to increase aerosolized mycobacterial numbers to be sent downwind toward the sentinels.

Regarding the assessment methods used in this study to examine human-like disease development and confirm transmission, we begin with the tuberculin skin test (TST). This test is one of the oldest diagnostic tests for tuberculosis. Although it can produce false results depending upon the immune and vaccination status of the individual, and relating the test result to the actual disease state is problematic, the test remains widely used due to ease of administration and low cost. In the initial infection study, various concentrations of PPD were tested along with Old Tuberculin, and induration was measured at 24, 48, and 72 hours. It was determined that 2 μg PPD injected intradermally with induration measured between 24-48 hours after injection was optimal. Using this testing format, all infected animals, regardless of low, medium, or high-infection dose, generated positive TST responses with induration of 12-25 mm. Concomitantly, in the initial transmission experiment where transmitter ferrets received a medium-high *M. tuberculosis* intratracheal dose, all converted to positive TST within 8 weeks. Interestingly, in the second transmission experiment where the transmitter ferrets received a very-high *M. tuberculosis* intratracheal dose, only two transmitters (TR-A1 and TR-A2) converted to TST positive at 4 weeks post infection. However, by 10 weeks post infection these two ferrets failed to respond to tuberculin, potentially demonstrating anergy due to an overwhelming infection (Delgado et al., 2002).

TST results in the sentinel ferrets also were quite interesting. In the medium-high infection dose experiment, only one of the seven sentinel animals, SE5 developed a positive TST result, but as indicated earlier, SE5, began co-housing with a transmitter 17 weeks post-infection, while all other sentinels were housed within 24 hours of transmitter infection. We hypothesize that this scenario enhances the likelihood of more efficient transmission. In the subsequent transmission study employing the very-high transmitter infection dose, likely because of the short duration of the exposure time with the transmitters (3 or 5 weeks), none of the sentinels developed positive TST results.

Expanding upon this point, although generally not converting their TST responses and generating low cytokine responses, the sentinels in the first transmission experiment that were placed with transmitters within 24 hours of infection carried viable bacilli and generated smaller non-caseous granulomas in their lungs. This type of TST and cytokine response discordance is routinely observed among human household contacts exposed to a culture-confirmed tuberculosis transmitter (Jones-López et al., 2015). A similar response was observed by Dharmadhikari and colleagues (2011) in guinea pigs naturally infected with *M. tuberculosis* bacilli directly from human patient coughs. As mentioned earlier, successful disease development depends on a number of factors including the dose of viable bacilli being transmitted and the duration of the exposure period (Beggs et al., 2003), with the standard diagnostic tests, such as TST, not able to delineate these aspects of the disease process. Our data support these observations.

Lastly, humans with high *M. tuberculosis* lung CFU have strong IgG antibody production and high inflammatory cytokine responses to mycobacterial antigens, whereas those that are asymptomatic typically are highly immune regulated (Kumar et al., 2013). Thus, immediately after infection, pro-inflammatory cytokines, such as IL-6 and IL-17, produced by the resident immune cells, upregulate key chemokines and adhesion molecules to recruit more leukocytes, which in turn are regulated by anti-inflammatory cytokines and regulatory T-cells. In our studies, this progression of responses was generally observed; however, even with high infection doses in the ferrets, variability was observed, which is often seen in genetically-diverse hosts. For example, in the transmission experiment employing transmitters given the very-high doses of *M. tuberculosis*, the data showed that lung expression of anti-inflammatory cytokines IL-4 and IL-10 was low in all transmitter ferrets that had to be euthanized early, whereas the two surviving transmitter animals euthanized at the end of the study had much higher expression of IL-10. However, expression of the gene encoding regulatory T cell FoxP3 was variable. In four of these transmitter ferrets, FoxP3 expression was high, while in ferret TR-A5, expression was negligible indicating an inability to control the inflammation. These FoxP3 responses require more study, as none of these six transmitter ferrets was able to control the infection. In addition to FoxP3, Myeloid Derived Suppressor Cells (MDSCs) may be responsible for ferrets’ inability to control infection. Higher MDSCs are usually accompanied by higher arginase levels, and the future studies will assess these levels (Guo et al., 2019). Although *M. tuberculosis* bacterial numbers in the lungs from the sentinels were not high enough to be cultured, the levels of IL-6, TNF-α and IL-1β were high in both aerosol and direct sentinels, indicating significant inflammation.

As described earlier, there are shortcomings with most assays used to assess tuberculosis disease progression in animal models, with some being specific to the model (e.g. no latency stage in mice) or shortage of immunological reagents, while other problems can be linked to the assays themselves. For example, in generating qualitative and quantitative data from clinical specimens, culturing and PCR methods are generally employed in small animal and human studies. However, to obtain a positive MGIT culture or direct viable plating count from a clinical specimen, 10-100 CFU/ml of bacteria are required in the collected specimen (Centers for Disease Control and Prevention, 2021). Thus, a positive culture is indicative of a significant infection in that particular tissue or fluid; however, this also illustrates that neither culturing clinical specimens by MGIT or direct plating is a highly-sensitive assay (Lessnau and Qarah, 2003; Mtafya et al., 2019). Although generally more sensitive than culture, qRT-PCR can be impaired by materials in the clinical samples, particularly from feces, which interfere with RNA purification or enzyme activities, invalidating or lessening the sensitivity of the assay (Caulfield and Wengenack, 2016). Thus, more than one culture method should be used to optimize the likelihood of detecting viable bacilli (Lessnau and Qarah, 2003), and qRT-PCR should also be considered. Nonetheless, as we demonstrated in this study, using a combination of available non-invasive assays increases the probability of identifying the presence of bacilli in the clinical specimens and, thus, providing a useful snapshot of the ongoing infection if not a maximally sensitive or specific diagnosis. Corroborating data on these observations can then be provided at necropsy.

## Conclusion

From the findings described above, we have established a tuberculosis acute disease model and *M. tuberculosis* transmission model using the ferret. There are a number of similarities between this model and the natural disease seen in humans, non-human primates, and other susceptible large mammals. Similarities include a natural resistance to infection, establishment of infection *via* likely both the aerosol and oral routes, spread of infection to the viscera, and in the potential to establish latent infection. These factors indicate that this model is pertinent to the study of tuberculosis and disease transmission in humans and the beginnings for understanding differences in the immune response that follow the development of active infection *via* natural transmission versus artificial infection.

## Data availability statement

The original contributions presented in the study are included in the article/**Supplementary Material**. Further inquiries can be directed to the corresponding author.

## Ethics statement

This study was conducted in accordance with the recommendations in the Guide for the Care and Use of Laboratory Animals and the American Veterinary Medical Association Guidelines for the Euthanasia of Animals. All animal experiments were performed with the approval of the Institutional Animal Care and Use Committee at the University of Georgia (NIH Animal Welfare Assurance Number: A3437-01) under protocol A2013 07-014-A1.

## Author contributions

Conceptualization: TG, CW, RK, ST, TR, FQ. Data curation: TG, NS, TR, ML, SHe, SO, TJ, SHa, WJ, TV, KS, FQ. Formal analysis: TG, NS, TR, TJ, KS, AB, FQ. Funding acquisition: FQ. Investigation: TG, NS, TR, ML, SHe, SO, TJ, SHa, WJ, TV, KS, CD, RK, FQ. Methodology: TG, NS, TR, ML, SHe, SO, TJ, SHa, WJ, TV, KS, CD, CW, RK, BH, ST, AB, TR, FQ. Project administration: TG, FQ. Resources: TG, NS, TR, ML, SHe, SO, TJ, SHa, WJ, TV, KS, CD, CW, RK, BH, ST, AB, TR, FQ. Software: TG, NS, TV, AB. Supervision: FQ. Validation: TG, NS, TR, SO, TJ, SHa, WJ, TV, KS, CD, CW, RK, BH, ST, AB, TR, FQ. Writing – original draft: TG, RK, FQ. Writing – review and editing: TG, NS, TR, ML, SHe, SO, TJ, SHa, WJ, TV, KS, CD, CW, RK, BH, ST, AB, TR, FQ. All authors contributed to the article and approved the submitted version.

## Funding

This study was funded by the UGA Interdisciplinary Proposal Development program.

## Acknowledgments

The authors are indebted to the staff of the UGA Animal Health Research Center and Animal Resources Program for their immense assistance in the maintenance and care of the ferrets. Sincere appreciation to Drs. James Posey and Melisa Willby from the Division of Tuberculosis Elimination, National Center for HIV/AIDS, Viral Hepatitis, STD, and TB Prevention, Centers for Disease Control and Prevention for their help with the MGIT analysis component of the study. The findings and conclusions in this report are those of the authors and do not necessarily represent the official position of the Centers for Disease Control and Prevention/Agency for Toxic Substances and Disease Registry.

## Conflict of interest

Author NS was employed by GlaxoSmithKline and TV was employed by Merck Research Laboratories.

The remaining authors declare that the research was conducted in the absence of any commercial or financial relationships that could be construed as a potential conflict of interest.

## Publisher’s note

All claims expressed in this article are solely those of the authors and do not necessarily represent those of their affiliated organizations, or those of the publisher, the editors and the reviewers. Any product that may be evaluated in this article, or claim that may be made by its manufacturer, is not guaranteed or endorsed by the publisher.
