# Supplementary material for: Ferrets as a model for tuberculosis transmission
Source: Front Cell Infect Microbiol. 2022 Aug 16;12:873416. doi: 10.3389/fcimb.2022.873416 (PMC9425069; doi:10.3389/fcimb.2022.873416)
Supplement: Supplementary file 1 [file DataSheet_1.pdf]

Gene	Designation	Sequence
CCL5	fCCL5 308 qf	CAGTTACCTTCGCAGTCCTC
	fCCL5 328 qR	AAATACTCCTGGATGTGGGTG
CXCL10	CXCL10 533 qF	ACGCTGTACCTGTATCAAGATC
	CXCL10 535 qR	TTCAGGCATCTTTTCTCCCC
IL-6	fIL6 551 qF	CAAATGTGAAGACAGCAAGGAG
	fIL6 553 qR	CCGGTAGTGATTCTTGTCAGAC
IFNγ	fIfNg 538 qf	GCCAAATTGTCTCCTTCTACTTG
	fIfNg 540 qR	GCTGCTGTTATTGAAGAACCTG
TNFα	ftnfa-F	5’-CCAGATGGCCTCCAACTAATCA-3’
	ftnfa-R	5’-GGCTTGTCACTTGGAGTTCGA-3’
IL-4	fIL4-F	5’-TCACCGGCACTTTCATCCA-3’
	fIL4-R	5’-TTCTCGCTGTGAGGATGTTCA-3’
IL-10	fIL10 248 qf	GAGAACCACGAGCCACAAG
	fIL-10 250 qR	ACCGCCTTGCTCTATCTC
FoxP3	ffoxp3 245 qf	GCGGACACTCAATGAGATCTAC
	ffoxp3 247 qR	CTTAGGTTGTGGCGATGG

Supplemental table 1. Cytokine primer sequences.

Suppl. 2A	High (5,000-10,000 CFU)						Control
Sample time	Week 4			Week 7			Weeks 4/7
Ferret #	1	2	3	4	5	6	19
Nasal wash	-	-	+	-	+	-	-/-
Throat swab	+	+	-	+	+	+	-/-
Trachea	-	+	+	+	+	-	-/-
Feces	-	NA	+	+	+	NA	-/-
Stomach	-	-	-	+	+	-	-/-
Lung	+	+	+	+	+	+	NA/-
Liver	NA	NA	NA	+	NA	NA	NA/-
Spleen	-	+	+	+	+	-	NA/-
TST	NA	NA	NA	+	+	+	NA/-

Suppl. 2B	Medium (100-200 CFU)						Low (10-50 CFU)						Control
Sample time	Week 4			Week 7			Week 4			Week 7			Weeks 4/7
Ferret #	7	8	9	10	11	12	13	14	15	16	17	18	19
Nasal wash	-	-	-	+	-	-	-	+	-	-	-	-	-/-
Throat swab	-	-	-	-	-	-	-	-	-	-	-	-	-/-
Trachea	-	+	+	+	NA	NA	-	-	-	-	+	-	-/-
Feces	-	NA	+	-	-	NA	-	NA	-	-	+	-	-/-
Stomach	-	-	-	-	+	-	-	-	-	-	-	-	-/-
Lung	+	+	+	+	+	+	+	+	+	+	+	+	NA/-
Liver	NA	NA	NA	NA	NA	NA	NA	+	NA	NA	+	NA	NA/-
Spleen	+	+	+	+	+	+	-	+	+	+	+	+	NA/-
TST	NA	NA	NA	+	+	+	NA	NA	NA	+	+	+	NA/-

Supplemental Table 2. Infection study: *M. tuberculosis* culture results from infected ferret samples.

Eighteen ferrets (six per dose) were infected by intratracheal instillation with *M. tuberculosis* strain Erdman with the indicated (A) high or (B) medium and low doses. At 4 and 7 weeks post-infection, specimens were collected. Nasal washes and throat swabs were obtained from lightly-anesthetized animals, and feces were collected from the cage pan housing two ferrets per cage. Lung, liver, stomach, trachea, and spleen tissues were harvested after euthanasia. For nasal wash, throat swab, trachea, feces, stomach and liver, (+) indicates positivity for presence of *M. tuberculosis* by MGIT culture with confirmation of *M. tuberculosis* DNA by IS6100 PCR. (-) indicates the absence of *M. tuberculosis* by MGIT and PCR. For lung and spleen homogenates, (+) indicates CFU detected after plating on agar while (-) indicates no CFU detected from any samples. For TST, in week 7, two days prior to euthanasia, skin tests were initiated by intradermal injection with purified protein derivative or Old Tuberculin and reactions assessed 24 hours later; a (+) TST result is defined as a diameter of induration > 12 mm. TST was only performed at the 7-week study termination point. NA indicates that a specific test or specimen was not assayed: Most samples from control ferret 19 were collected at both time points except for tissue samples collected at study termination.

Suppl. 3A<br>Sample time	27 weeks post infection				27 weeks post co-housing			4 weeks post co-housing		10 weeks post co-housing	Weeks 17/27 Controls	
Ferret #	TR1	TR2	TR3	TR4	SE1	SE2	SE3	SE6	SE7	SE5	9	10
Nasal wash	+	+	-	+	+	+	+	+	-	-	-/-	-/-
BAL	-	+	+	+	-	-	-	-	-	+	-/-	-/-
Lung	+	+	+	+	+	+	+	+	+	+	NA/-	NA/-
Spleen	+	+	+	+	+	+	+	-	+	+	NA/-	NA/-
Liver	+	-	-	-	+	-	-	-	-	-	NA/-	NA/-
TST	+	+	+	+	-	-	-	-	-	+	-/-	-/-

Suppl. 3B<br>Sample time	Week 5 post infection		Week 8 post infection		Week 10 post infection*		Week 3 post co-housing	Week 5 post co-housing		Week 3 post co-housing		Week 5 post co-housing				Weeks 4/10 Controls	
Ferret #	TR A4	TR A5	TR A3	TR A6	TR A1	TR A2	SE D3	SE D1	SE D2	SE A3	SE A6	SE A1	SE A2	SE A4	SE A5	15	16
Nasal wash	+	+	-	+	-	+	+	+	+	-	-	-	-	-	+	NA	NA
Trachea	-	+	-	+	+	+	-	-	-	-	-	+	-	-	-	NA/-	NA/-
Lung	+	+	+	+	+	+	-	-	-	-	-	-	-	-	-	NA/-	NA/-
Lymph node	+	+	+	+	+	+	-	-	-	-	-	-	-	-	-	NA/-	NA/-
Spleen	+	+	+	+	+	+	-	-	-	-	-	-	-	-	-	NA/-	NA/-
Liver	+	+	+	+	+	+	-	-	-	-	-	-	-	-	-	NA/-	NA/-
TST	-	-	-	-	+/-	+/-	-	-	-	-	-	-	-	-	-	-/-	-/-

Supplemental Table 3. Transmission studies: Results from *M. tuberculosis*-infected transmitter and co-housed sentinel ferrets.

(A) Transmitter (TR) ferrets were intratracheally infected with a medium-high dose ($1X10^3$ CFU) of *M. tuberculosis* Erdman. Samples were collected from the TR ferrets 27 weeks post infection, and from sentinel (SE) ferrets 4, 10, or 27 weeks after co-housing with the respective TR ferrets. Nasal washes and BAL were obtained from lightly-anesthetized animals. Lung, spleen and liver tissues were harvested after euthanasia. (+) indicates positive nasal washes for presence of *M. tuberculosis* DNA by IS6100 PCR, and in BAL by MGIT culture and IS6100 PCR. For lung and spleen homogenates, (+) indicates CFU detected after plating on agar; liver homogenates were assessed by MGIT culture and PCR. (-) indicates the absence of *M. tuberculosis* by all described tests. TST was assessed every four to five weeks in TR and SE animals during the course of the study with the final test two days prior to study termination (see Figure 5); the controls were assessed at 17 and 27 weeks post-TR infection time points.
(B) Transmitter (TR) ferrets were intratracheally infected with a very-high dose ($5 X 10^4$ CFU). Samples were collected from TR ferrets 10 weeks post-infection (or when humane end points were reached), and from SE ferrets 3 and 5 weeks after co-housing. Nasal washes were collected from anesthetized animals. Trachea, lung, mediastinal lymph nodes, spleen, and liver assessments were made after necropsy. For nasal wash and trachea, (+) indicates positivity for presence of *M. tuberculosis* by IS6100 PCR, while for lung, lymph node, spleen and liver homogenates, (+) indicates CFU detected after plating on agar. TST was assessed two days prior to study termination time points; for the controls, the transmitter 4 and 10 week post-infection time points were used. *****TST results for TR-A1 and TR-A2 were (+) at the 4 week time point and (-) at study termination. For both tables, a (+) TST result is a diameter of induration > 12 mm. NA indicates that a specific test or specimen was not assayed.

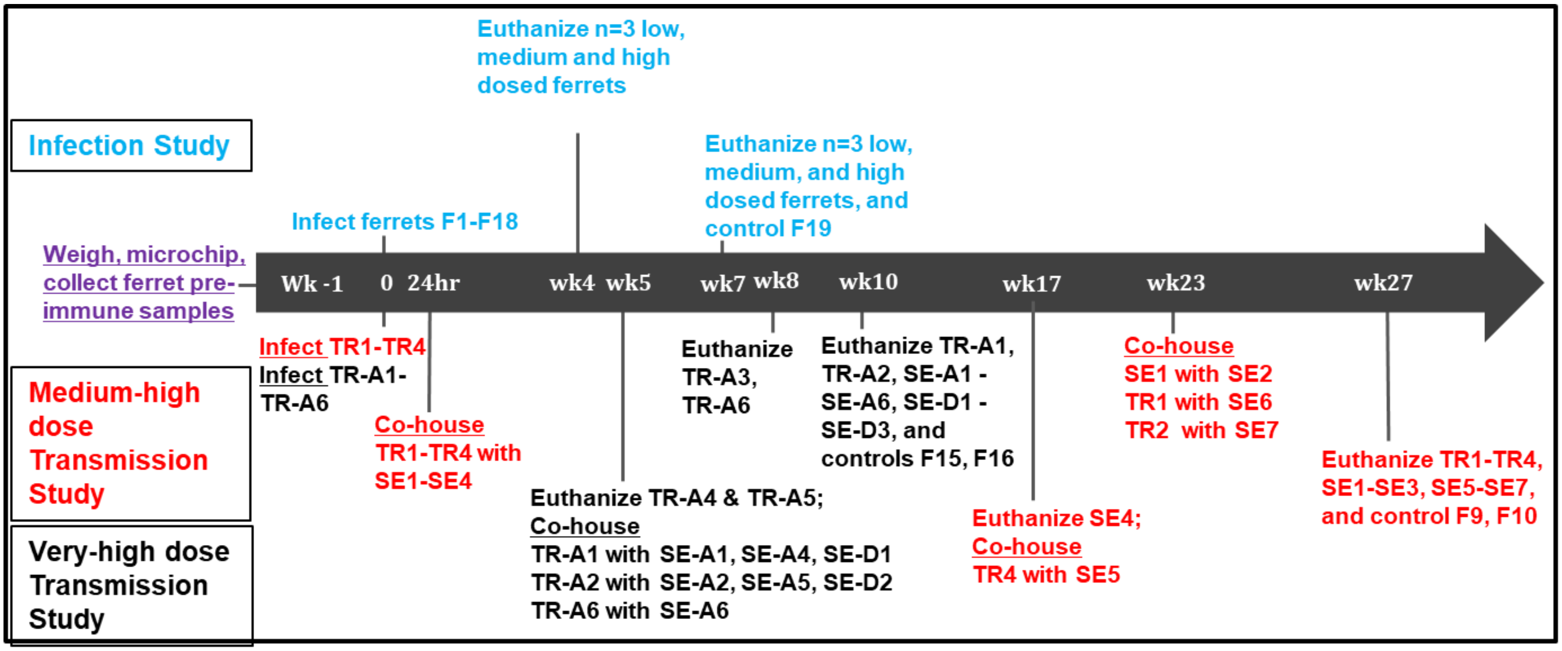

Supplemental figure 1: Timeline for (top) Infection and (bottom) transmission studies. See Table 1 for the types and times of non-invasive testing performed in these studies.

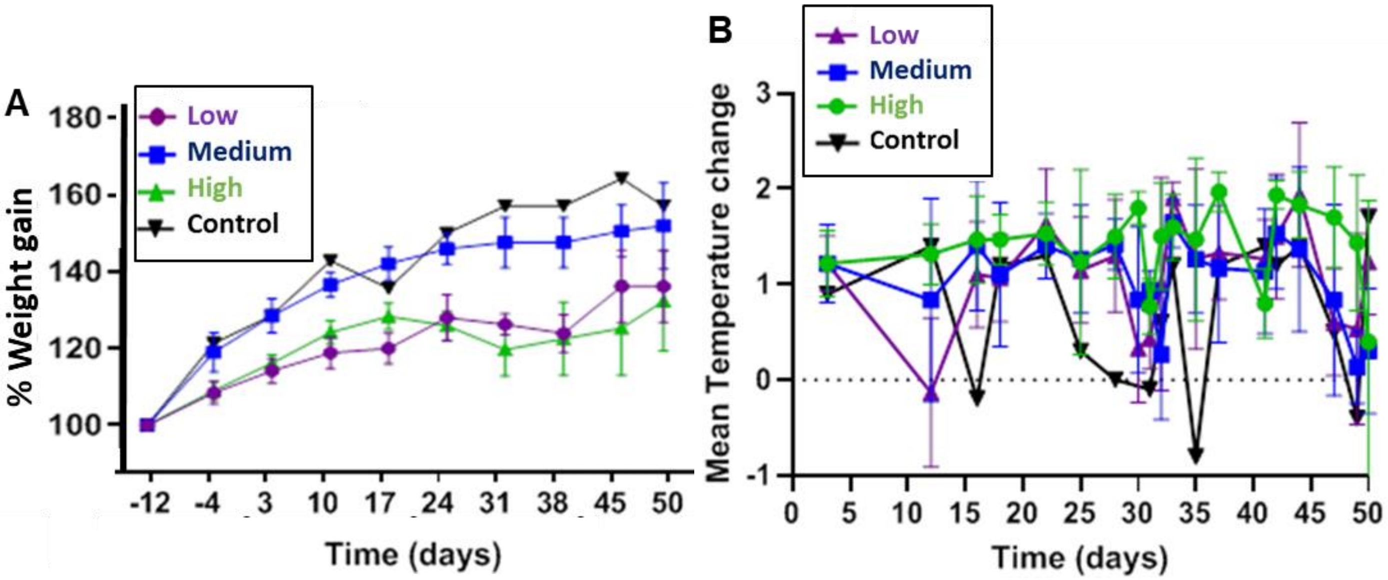

Supplemental figure 2. Infection study weight and temperature changes: A) Changes in weight gained among *M. tuberculosis*-infected ferret groups. In this study, ferrets were intratracheally infected with a low (10 - 50), medium (100 - 500), or high (5,000 – 10,000) CFU dose of *M. tuberculosis*, and the weight of the infected animals and an uninfected control ferret was measured over seven weeks. For each *M. tuberculosis* dose group, the mean percentage weight change with standard error was based on n = 6 animals prior to day 28 and n = 3 after day 28. B) Temperature change in *M. tuberculosis*-infected ferrets. Ferret temperatures were monitored a minimum of once per week. The changes in temperature (°C) for each time point were calculated by subtracting the mean of the baseline temperature (days -7 to 0) for each animal group from the mean temperature observed at the indicated time points for the same group of animals.

A

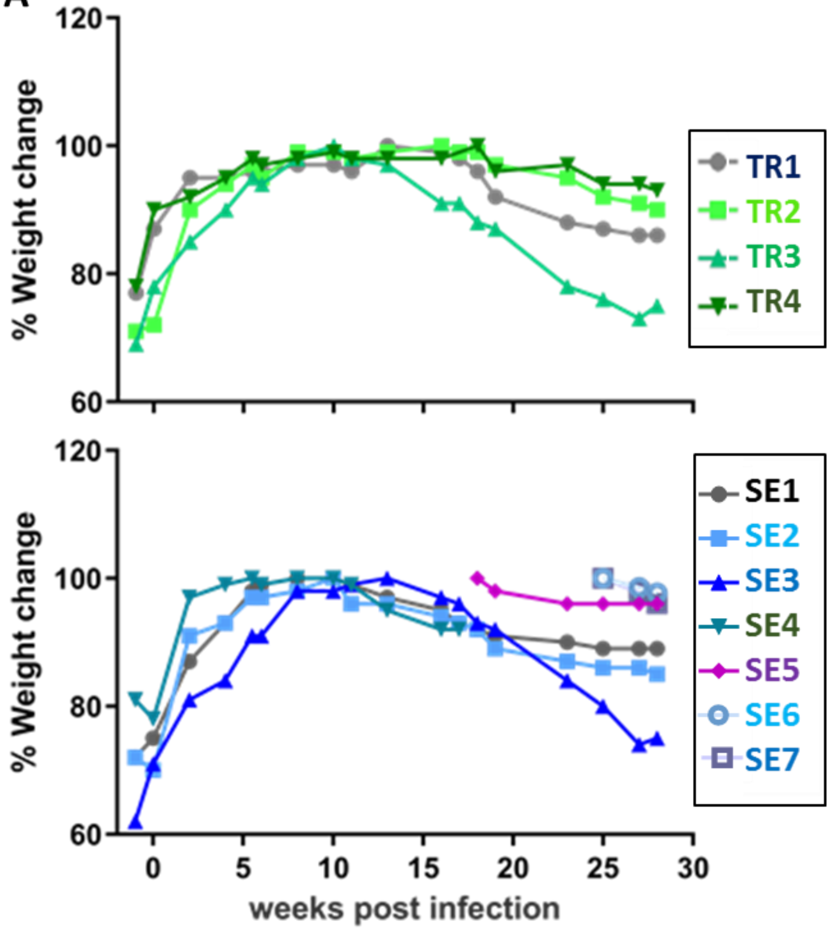

120
100
80
60
% Weight change
TR1
TR2
TR3
TR4
120
100
80
60
% Weight change
0
5
10
15
20
25
30
weeks post infection
SE1
SE2
SE3
SE4
SE5
SE6
SE7

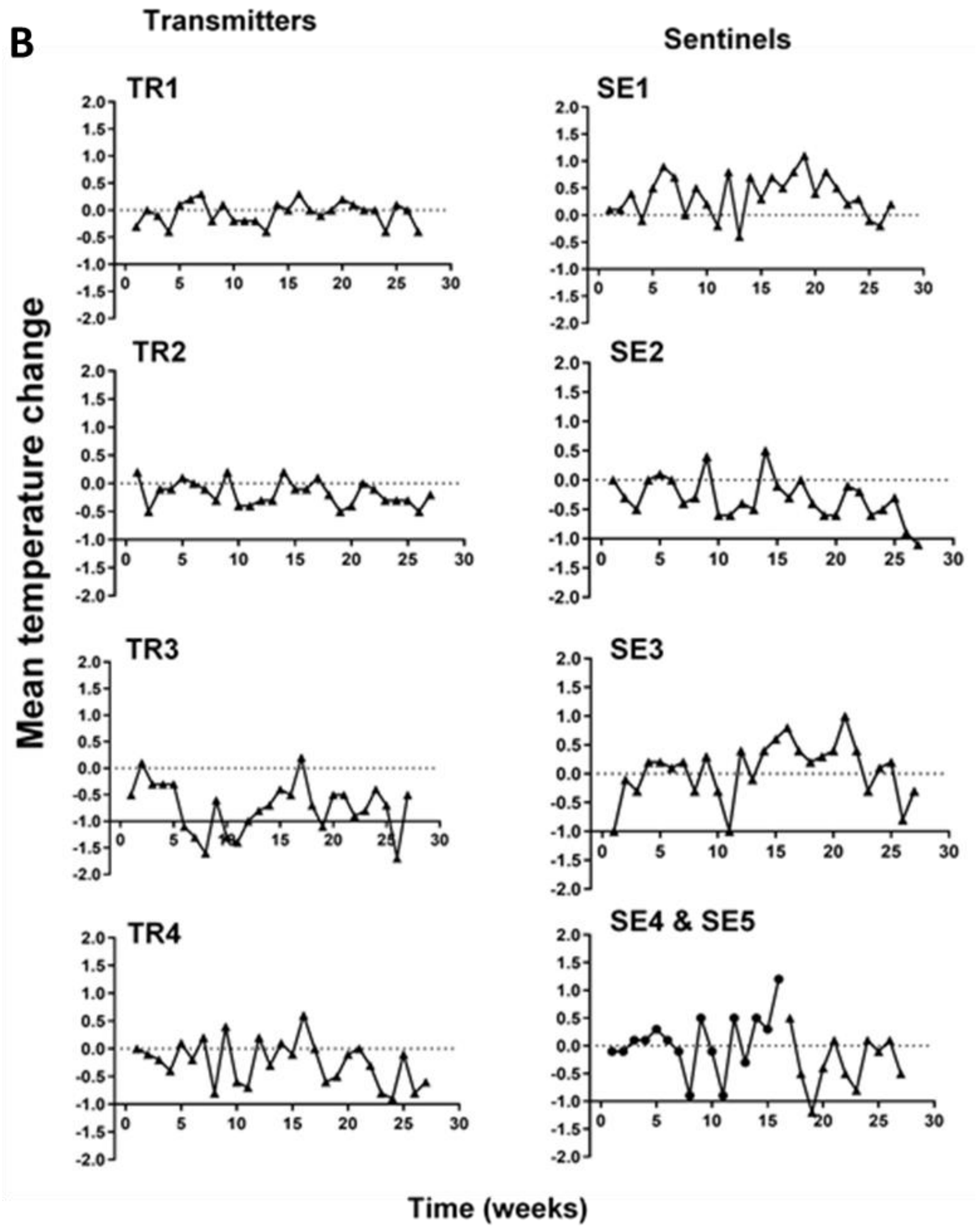

Supplemental figure 3. Transmission study using a medium-high dose infected transmitters: A) Weight change in transmitters and sentinels. Transmitter (upper panel) and sentinel (lower panel) ferret weights were monitored. Percent weight change was calculated as a function of maximal weight attained by each animal [(weekly weight/maximum weight)*100]. Sentinel ferrets 1-4 were placed with transmitter counterparts 24 hours after transmitters were infected. Sentinels 5-7 were placed with transmitters later in study. B) Temperature spikes in transmitters and sentinels. Ferret temperatures were monitored five days per week. The changes in temperature (°C) for each time point were calculated by subtracting the mean of the baseline temperature (days -7 to 0) for each animal from the maximum temperature observed in the corresponding animal each week. In the panel on the lower right, temperatures for SE4 (●) and SE5 (▲) are shown.

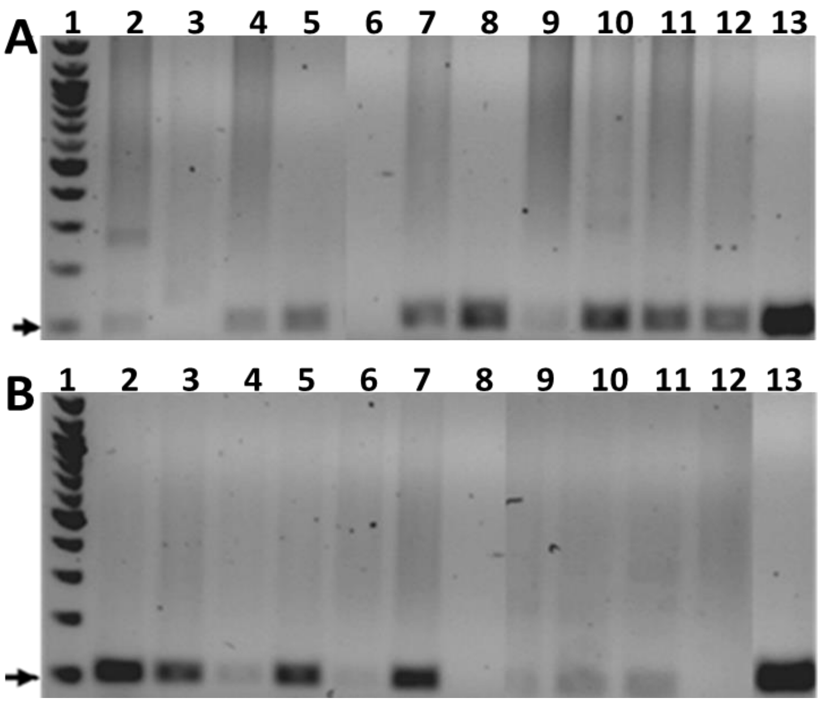

Supplemental figure 4. Transmission study using medium-high dose infected transmitters: IS6110 PCR of ferret lung and spleen samples. Shown are composite gel images of IS6110 PCR products from lung (panel A) and spleen (panel B) homogenates from the indicated transmitter (TR) or sentinel (SE) ferrets resolved on a 2% agarose gel. The arrows indicate the expected band size corresponding to the 106 bp IS6110 amplicon. A) Lane 1 = 100 bp ladder (NEB), 2 = TR1, 3 = TR2, 4 = TR3, 5 = TR4, 6 = negative control DNA, 7 = SE1, 8 = SE2, 9 = SE3, 10 = SE5, 11 = SE6, 12 = SE7, and 13 = 106 bp fragment from the positive-control DNA. B) Lane 1 = 100 bp ladder (NEB), 2 = SE1, 3 = SE2, 4 = SE3, 5 = SE5, 6 = SE6, 7 = SE7, 8 = negative control DNA, 9 = TR1, 10 = TR2, 11 = TR3, 12 = TR4, and 13 = positive control amplified with *M. tuberculosis* genomic DNA template. The samples were collected at study termination. Panel A is a composite image of two gels run simultaneously. The molecular weight marker lanes for the two gels aligned perfectly.

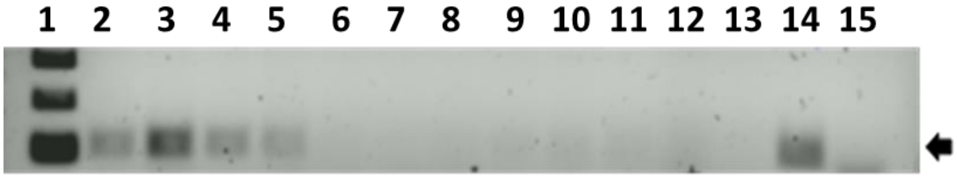

Supplemental figure 5. IS6110 PCR of cage plenum Shown is a gel image of IS6110 PCR products from cage plenum resolved on a 2% agarose gel. The arrows indicate the expected band size corresponding to the 106 bp IS6100 amplicon. Each cage plenum contains 5 segments. Data for each cage are shown as swabs taken from one plenum segment (cage #) or all plenum segments combined (pooled cage #). Lane 1 = 100 bp ladder (NEB), 2 = pooled cage 1, 3 = cage 1, 4 = pooled cage 2, 5 = cage 2, 6 = pooled cage 3, 7 = cage 3, 8 = pooled cage 4, 9 = cage 4. 10 = pooled cage 5, 11 = cage 5, 12 = pooled cage 6, 13 = cage 6, 14 = positive control, 15= negative control. The cage numbers correlate with the transmitter and sentinel animal numbers, e.g. cage 1 contained TR-A1, SE-D1 and SE-A1. The samples were collected at study termination.

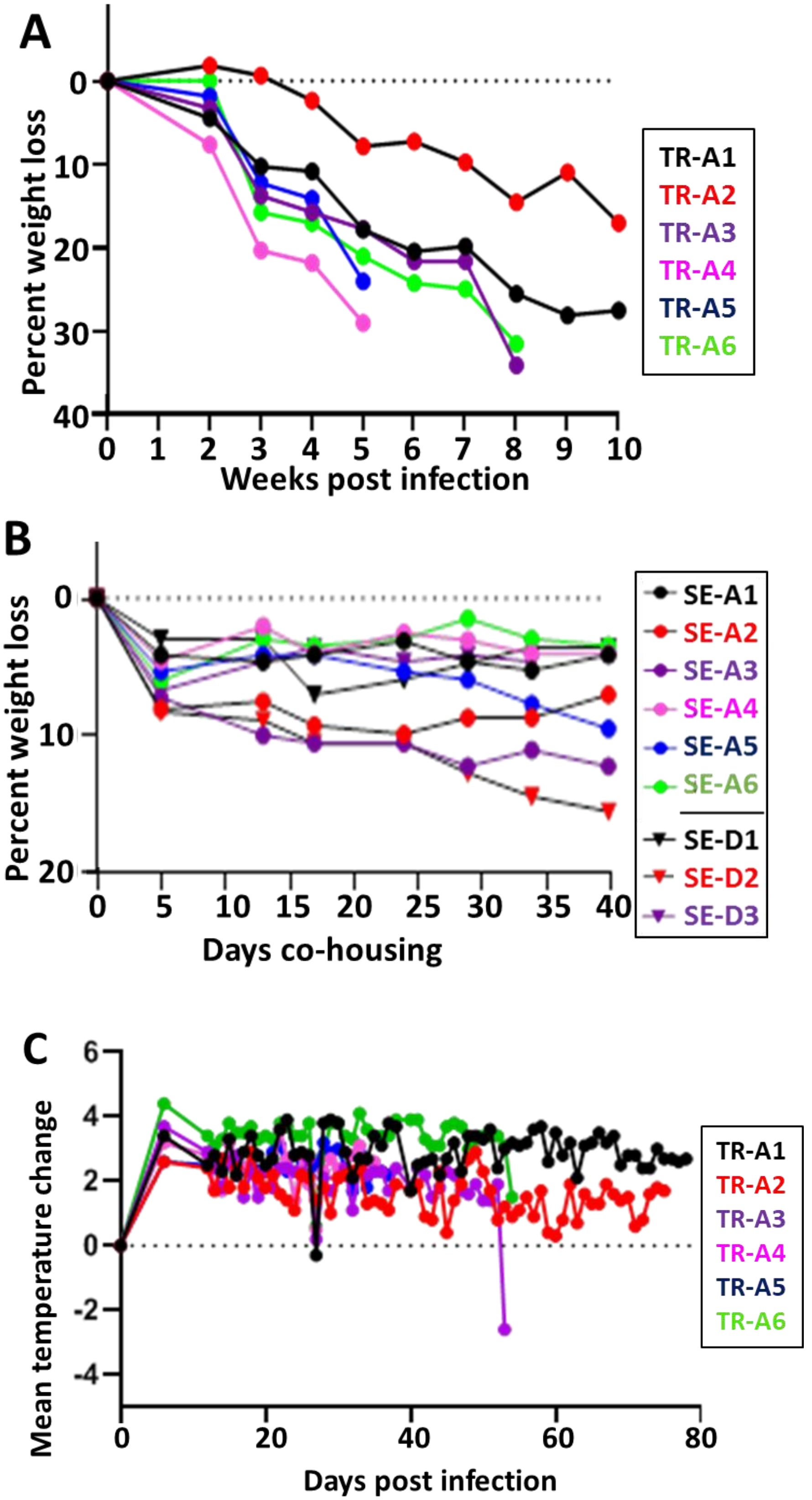

A
Percent weight loss
Weeks post infection
TR-A1
TR-A2
TR-A3
TR-A4
TR-A5
TR-A6
0
10
20
30
40
0 1 2 3 4 5 6 7 8 9 10
B
Percent weight loss
Days co-housing
SE-A1
SE-A2
SE-A3
SE-A4
SE-A5
SE-A6
SE-D1
SE-D2
SE-D3
0
10
20
0 5 10 15 20 25 30 35 40
C
Mean temperature change
Days post infection
TR-A1
TR-A2
TR-A3
TR-A4
TR-A5
TR-A6
6
4
2
0
-2
-4
0 20 40 60 80

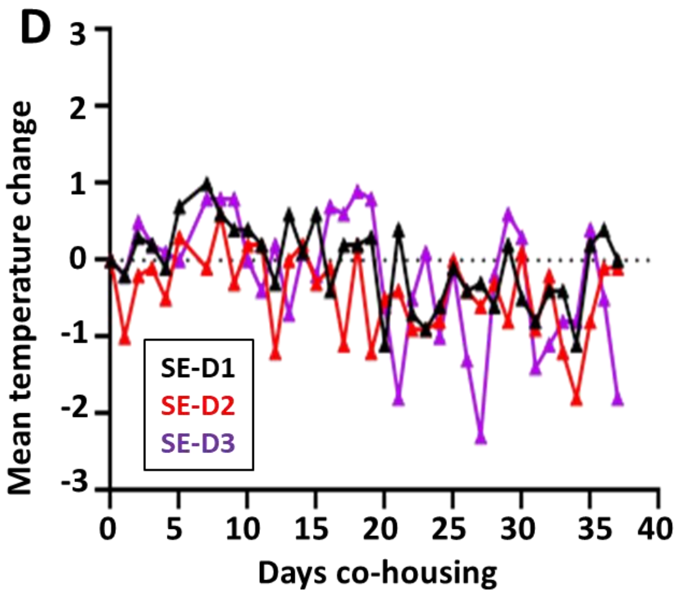

Supplemental figure 6. Transmission study using very-high dose-infected transmitters: Weight loss and temperature change in transmitters and sentinels. Ferret weights were measured daily from 1 week prior to infection until the end of the 10-week study. The weekly average percent weight loss relative to the pre-study weight difference (dotted line) is plotted for the transmitter (A) and sentinel (B) ferrets. Ferret temperatures were monitored daily. The changes in temperature (°C) for each time point were calculated by subtracting the mean of the baseline temperature (days -7 to 0) for each animal from the temperature observed each day for the transmitter (C) and direct-sentinel (D) ferrets.

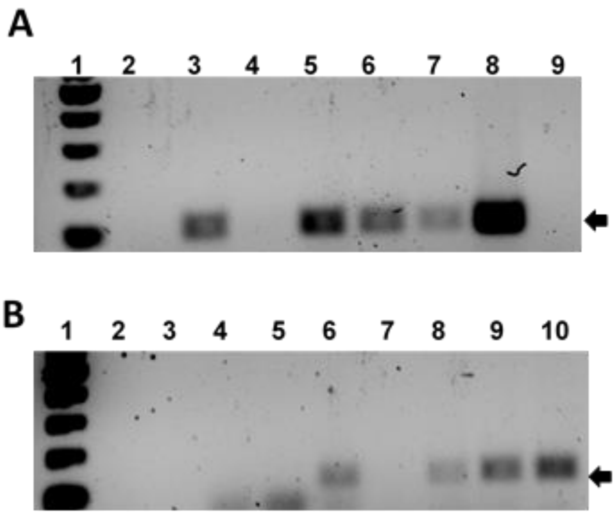

Supplemental figure 7. Transmission study using very-high dose infected transmitters: IS6110 PCR of ferret nasal washes. Shown are gel images of IS6110 PCR products from nasal washes resolved on a 2% agarose gel. The arrows indicate the expected band size corresponding to the 106 bp IS6100 amplicon A) Lane 1 = 100 bp ladder (NEB), 2 = TR-A1, 3 = TR-A2, 4 = TR-A3, 5 = TR-A4, 6 = TR-A5, 7 = TR-A6, 8 = positive control DNA, 9 = negative control DNA. B) Lane 1 = 100 bp ladder, 2 = SE-A1, 3 = SE-A2, 4 = SE-A3, 5 = SE-A4, 6 = SE-A5, 7 = SE-A6, 8 = SE-D1, 9 = SE-D2, 10 = SE-D3. The transmitter samples were collected at week 4 post infection, and the sentinel samples were collected at week 4 of co-housing with the transmitters.
